# VCF2CNA: A tool for efficiently detecting copy-number alterations in VCF genotype data and tumor purity

**DOI:** 10.1038/s41598-019-45938-x

**Published:** 2019-07-17

**Authors:** Daniel K. Putnam, Xiaotu Ma, Stephen V. Rice, Yu Liu, Scott Newman, Jinghui Zhang, Xiang Chen

**Affiliations:** Department of Computational Biology, St. Jude Children’s Research Hospital, Memphis, TN USA

**Keywords:** Cancer genomics, Software

## Abstract

VCF2CNA is a tool (Linux commandline or web-interface) for copy-number alteration (CNA) analysis and tumor purity estimation of paired tumor-normal VCF variant file formats. It operates on whole genome and whole exome datasets. To benchmark its performance, we applied it to 46 adult glioblastoma and 146 pediatric neuroblastoma samples sequenced by Illumina and Complete Genomics (CGI) platforms respectively. VCF2CNA was highly consistent with a state-of-the-art algorithm using raw sequencing data (mean F1-score = 0.994) in high-quality whole genome glioblastoma samples and was robust to uneven coverage introduced by library artifacts. In the whole genome neuroblastoma set, VCF2CNA identified MYCN high-level amplifications in 31 of 32 clinically validated samples compared to 15 found by CGI’s HMM-based CNA model. Moreover, VCF2CNA achieved highly consistent CNA profiles between WGS and WXS platforms (mean F1 score 0.97 on a set of 15 rhabdomyosarcoma samples). In addition, VCF2CNA provides accurate tumor purity estimates for samples with sufficient CNAs. These results suggest that VCF2CNA is an accurate, efficient and platform-independent tool for CNA and tumor purity analyses without accessing raw sequence data.

## Introduction

Copy-number alterations (CNAs) are gains or losses in chromosomal segments that frequently occur in tumor cells. Recent surveys suggest that certain cancers are driven by CNAs^1^. In addition to directly affecting cancer genes (e.g., *MYCN* and *MDM2* amplifications and *RB1* and *CDKN2A* deletions), CNAs are known to be driver events in a wide variety of cancer types^2–5^. Several experimental methods are available to identify CNAs in tumor cells. Fluorescence *in situ* hybridization provides direct evidence of CNAs and is the gold standard for CNA detection in a targeted region^6^. Before the development of next-generation sequencing (NGS) technologies, array comparative genomic hybridization and high-resolution single nucleotide polymorphism (SNP) arrays permitted genome-wide evaluation of CNAs at 30-kb to 100-kb resolution.

The development of NGS, especially whole-genome sequencing (WGS) and whole-exome sequencing (WXS) platforms, has revolutionized the detection of somatic mutations, including CNAs, in cancer samples. For example, Copy Number Segmentation by Regression Tree in Next Generation Sequencing (CONSERTING)^7^ incorporates read-depth and structural-variation data from BAM files for accurate CNA detection in high-coverage WGS data. However, CONSERTING and other WGS-based CNA algorithms produce a fractured genome pattern (i.e., a hypersegmented CNA profile with an excessive number of intrachromosomal translocations) in samples with library construction artifacts^7^, which poses a major challenge for precise CNA inference. Although the frequency of observing the fracture genome pattern has dropped substantially with improvement of libraries preparation and sequencing methods, we still occasionally identify the pattern in samples sequenced with the latest technology. Our extensive analysis indicated that although CNA and structural-variation detection was severely impaired by library artifacts, point-mutation detection was largely unaffected^7,8^, suggesting that a robust CNA tool can be developed from the variant information. CONSERTING and other NGS algorithms require direct access to aligned BAM files. Moreover, advances in technology and declines in costs have made NGS a commodity for both basic research and clinical service. However, local installation of CONSERTING and other NGS algorithms often involves complicated steps, which may be challenging for individual groups without dedicated bioinformatics support. Cloud-based pipelines may require transfer of large BAM files, a current bottleneck in their applications. Therefore, a robust CNA analytical tool that is efficient, convenient, and robust to library artifacts is needed to manage the demands of NGS data analysis.

VCF2CNA is both a web-based (http://vcf2cna.stjude.org), and commandline (http://www.github.com/XCLab/VCF2CNA) tool for CNA analysis. The preferred input to VCF2CNA is a paired Tumor/Germline Variant Call Format (VCF) file. VCF is a widely adopted format for genetic variation data exchange, and VCF files are quite small compared to WGS BAM files. Each variant in a typical VCF file contains its chromosome position, reference/alternative alleles, and corresponding allele counts, which are used by VCF2CNA to identify copy-number alterations. This tool also accepts input in the Mutation Annotation Format (MAF) and the variant file format produced by the Bambino program^9^.

## Results

**Figure 1:**
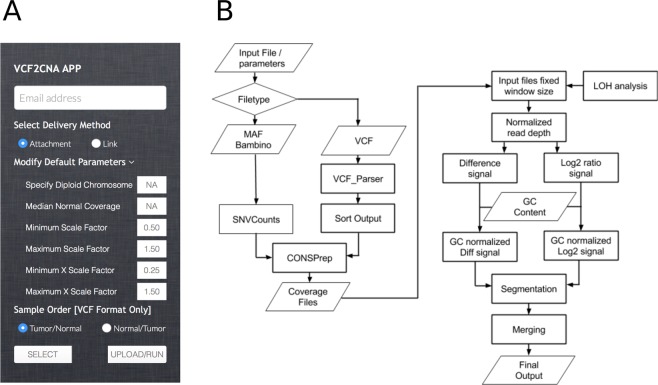
Overview of the VCF2CNA process. (**A**) User interface with parameters. (**B**) Server side pipeline. A parallelogram depicts input or output files, a rectangle depicts an analytical process, and a diamond depicts the condition for a follow-up process.

VCF2CNA can be run through a simple web interface (Fig. 1A) or as a commandline line tool. For the web interface, the sole input is a VCF file (or a file in one of the other supported variant file formats) from a paired tumor–normal WGS or WXS analysis, which is uploaded via the interface to a web server where the application runs. The results are returned to a user-provided email address. For the commandline tool, the pipeline is run by invoking a single run command. VCF2CNA consists of two main modules: (1) SNP information retrieval and processing from the input data and (2) recursive partitioning–based segmentation using SNP allele counts (Fig. 1B). Actual running time for a typical WGS sample is approximately 30 to 60 minutes, depending on the complexity of the genome.

To evaluate the utility of VCF2CNA, we ran it on 192 tumor–normal WGS data sets and 15 tumor–normal WXS data sets. These sequences comprised 46 WGS adult glioblastomas (GBMs) from The Cancer Genome Atlas (TCGA-GBM) dataset^10^, sequenced by Illumina technology, and 146 WGS pediatric neuroblastomas (NBLs) from the Therapeutically Applicable Research to Generate Effective Treatments (TARGET-NBL) dataset^11^, sequenced by Complete Genomics, Inc. (CGI) technology. On average, VCF2CNA used approximately 2.8 million high-quality SNPs per sample (median 2,811,245; range, 2,029,467–3,519,454 in TARGET-NBL data) to derive CNA profiles. We further evaluated the consistency between WGS and WXS using 15 rhabdomyosarcoma samples that were sequenced on both platforms^12^ and estimated the tumor purity in these samples.

### CNA analysis of TCGA-GBM data

**Figure 2:**
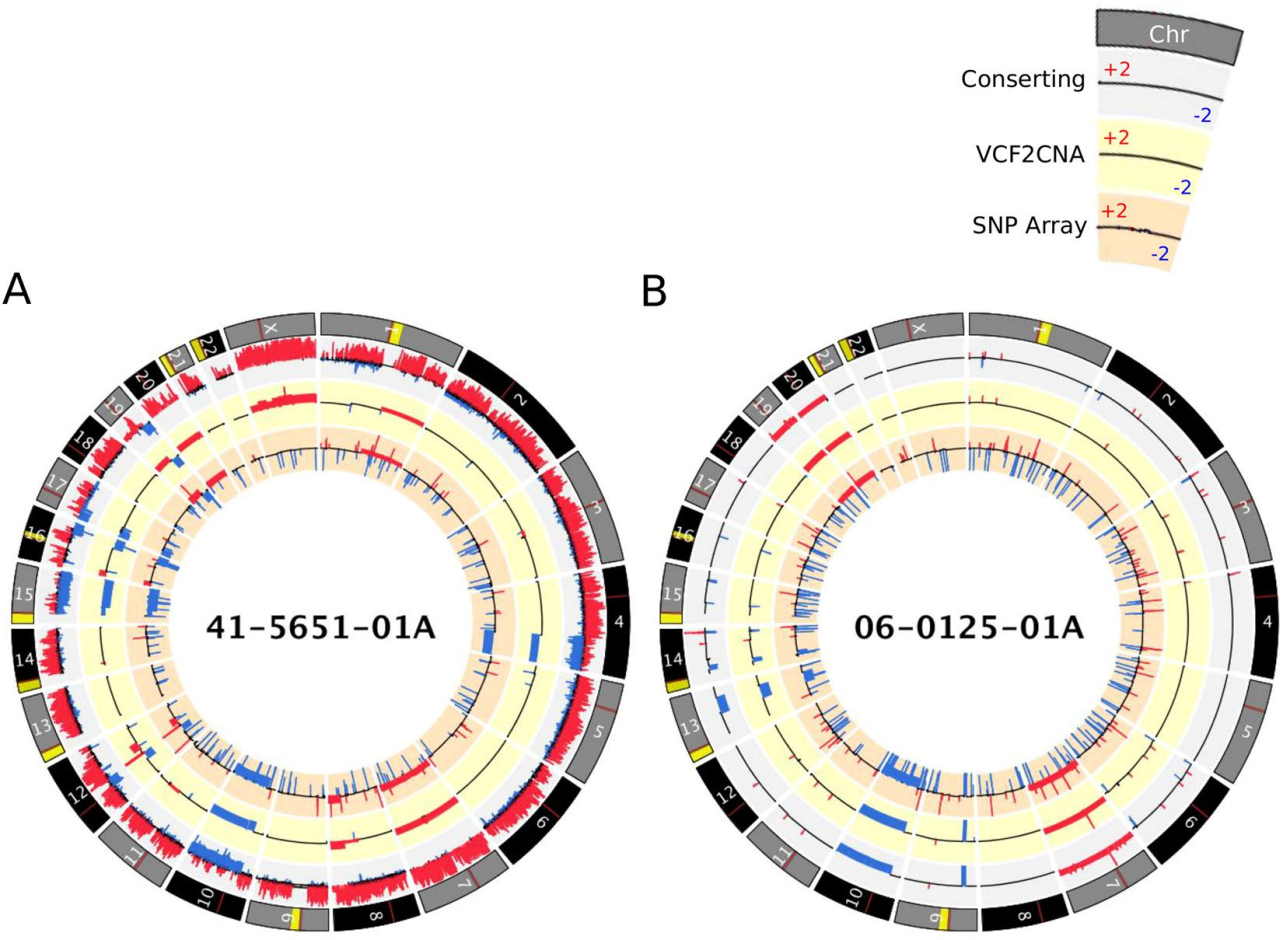
A Circos plot that displays CNAs found by CONSERTING (outer ring), VCF2CNA (middle ring), and SNP array (inner ring) for (**A**) TCGA-GBM fractured sample 41-5651-01A and (**B**) TCGA-GBM unfractured sample 06-0125-01A. Alternating gray and black chromosomes are used for contrast. Yellow regions depict sequencing gaps, whereas red regions depict centromere location. Blue segments depict copy-number loss, and red segments indicate copy-number gain. Legend depicts CNA range for each track.

The adult TCGA-GBM data downloaded from dbGaP (accession number: phs000178.v8.p7) included 46 samples. We first evaluated VCF2CNA’s resistance to library construction artifacts by using 24 samples from this set, which were previously identified as having a fractured genome pattern by CONSERTING and other CNA algorithms^7^. Indeed, VCF2CNA produced CNA profiles that are globally consistent with those of SNP array–derived CNA profiles (downloaded from TCGA, Supplementary File s1) and more robust to noise than those produced by CONSERTING. Specifically, VCF2CNA yielded a mean 59.4-fold reduction in the number of predicted segments than did CONSERTING (median, 46.2; range, 16.2–285.7; *p* = 3.0 × 10^−6^ by Wilcoxon signed-rank test, Fig. 2A and Supplementary File s1).

We used an F_1_ scoring metric^13^ to measure the consistency between the CNA profiles derived from VCF2CNA and CONSERTING in the remaining 22 high-quality sample pairs (Fig. 2B and Supplementary File s2). These programs identified approximately 700 Mb of the CNA regions in each sample (range, 92–2299 Mb) with high consistency (mean F_1_ score, 0.9941; range, 0.9699–0.9995) (Table 1).

**Table 1:** F_1_ score between CONSERTING and VCF2CNA and autosomal CNAs per sample in 22 TCGA samples.

Sample	F_1_ score	Autosomal CNAs per sample (Mb)
SJHGG011906_D1_G1_N13	0.9699	567.70
SJHGG010485_D1_G1	0.9840	789.90
SJHGG011903_D1_G1	0.9862	459.20
SJHGG010643_D1_G1_N5	0.9870	1471.67
SJHGG010641_D1_G1	0.9884	285.89
SJHGG010600_R1_G1	0.9892	485.85
SJHGG010484_R1_G1_N2	0.9949	2299.48
SJHGG010560_R1_G1	0.9955	756.08
SJHGG010624_R1_G1	0.9956	1259.68
SJHGG010600_D1_G1	0.9968	389.60
SJHGG010485_R1_G1	0.9970	92.16
SJHGG011904_D1_G1	0.9979	696.48
SJHGG010540_D2_G1	0.9981	660.74
SJHGG010484_D1_G1	0.9983	841.72
SJHGG010509_D1_G1	0.9983	586.18
SJHGG010560_D1_G1	0.9984	551.73
SJHGG010577_D1_G1	0.9984	831.67
SJHGG010509_R1_G1	0.9988	562.44
SJHGG010572_R1_G1	0.9992	427.91
SJHGG010572_D1_G1	0.9994	456.27
SJHGG010624_D1_G1	0.9995	454.09
SJHGG010540_R1_G1	0.9995	463.89

**Figure 3:**
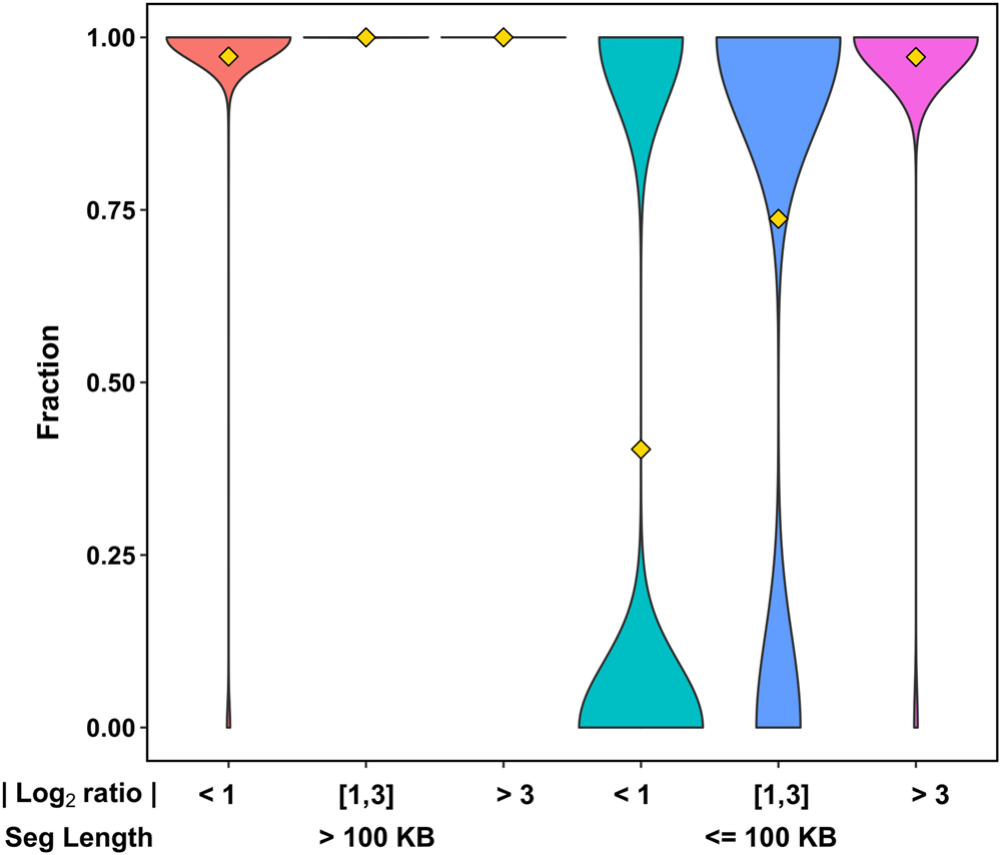
Violin plot stratified by segment size and CNA intensity for all 22 TCGA-GBM unfractured samples. Gold diamond represents the mean fraction of matching segments between VCF2CNA and CONSERTING.

We evaluated the segmental overlap between the CONSERTING outputs and the VCF2CNA outputs for each sample. A CNA segment detected by CONSERTING was classified as corroborated if 90% of the bases in the segment received the same type of CNA call from VCF2CNA (Table 2). The comparison shows that VCF2CNA faithfully recapitulated medium to large CNA segments (≥100 kb), whereas CONSERTING had greater power for identifying focal (<100 kb) low-amplitude (absolute log2 ratio change <1.0) CNAs (*p* = 1.306 × 10^−5^ by Wilcoxon signed-rank test). Furthermore, the segmental–based analysis revealed that the detection power was less affected in focal CNAs with large amplitudes (log2 ratio ≥ 3.0) (Fig. 3).

**Table 2:** Counts of corroborated and uncorroborated segments by segment length.

Sample	Matched segment length (log_10_)					Unmatched segment length (log_10_)					Match percentage	
	<3	[3,4)	[4,5)	[5,6)	>6	<3	[3,4)	[4,5)	[5,6)	>6	<100 kb	≥100 kb
SJHGG010484_D1_G1	0	4	45	24	54	2	9	31	3	0	0.5385	0.9630
SJHGG010484_R1_G1A	4	8	23	21	90	8	7	3	1	0	0.6604	0.9911
SJHGG010485_D1_G1	8	5	20	20	40	20	25	16	4	1	0.3511	0.9231
SJHGG010485_R1_G1	0	0	0	0	2	9	1	3	1	0	0.0000	0.6667
SJHGG010509_D1_G1	3	0	9	15	24	3	0	5	0	0	0.6000	1.0000
SJHGG010509_R1_G1	5	0	11	11	24	8	1	4	1	0	0.5517	0.9722
SJHGG010540_D2_G1	5	11	46	25	28	4	10	5	0	0	0.7654	1.0000
SJHGG010540_R1_G1	4	9	31	32	22	3	11	9	0	0	0.6567	1.0000
SJHGG010560_D1_G1	9	30	59	32	20	24	39	20	0	0	0.5414	1.0000
SJHGG010560_R1_G1	2	0	5	17	26	24	25	15	3	1	0.0986	0.9149
SJHGG010572_D1_G1	2	5	23	27	26	38	12	7	0	0	0.3448	1.0000
SJHGG010572_R1_G1	2	2	4	24	18	30	18	8	1	0	0.1250	0.9767
SJHGG010577_D1_G1	7	4	24	36	37	15	12	9	2	0	0.4930	0.9733
SJHGG010600_D1_G1	29	26	45	79	32	40	26	17	1	0	0.5464	0.9911
SJHGG010600_R1_G1	18	26	50	65	27	51	28	11	2	0	0.5109	0.9787
SJHGG010624_D1_G1	13	13	114	53	82	32	7	2	0	0	0.7735	1.0000
SJHGG010624_R1_G1	9	8	143	110	202	22	4	17	1	0	0.7882	0.9968
SJHGG010641_D1_G1	27	50	99	62	39	19	325	175	3	0	0.2532	0.9712
SJHGG010643_D1_G1B	5	13	22	33	30	24	24	11	15	2	0.4040	0.7875
SJHGG011903_D1_G1	1	0	4	39	13	2	5	0	0	1	0.4167	0.9811
SJHGG011904_D1_G1	1	2	4	14	23	1	1	5	1	0	0.5000	0.9737
SJHGG011906_D1_G1C	3	14	42	26	44	10	19	27	3	0	0.5130	0.9589
SJHGG010484_D1_G1	0	4	45	24	54	2	9	31	3	0	0.5385	0.9630

The baseline (diploid) signal was automatically inferred from VCF2CNA for all but three samples. User specified baseline signal: A) chromosome 2, B) chromosome 5, C) chromosome 13.

**Figure 4:**
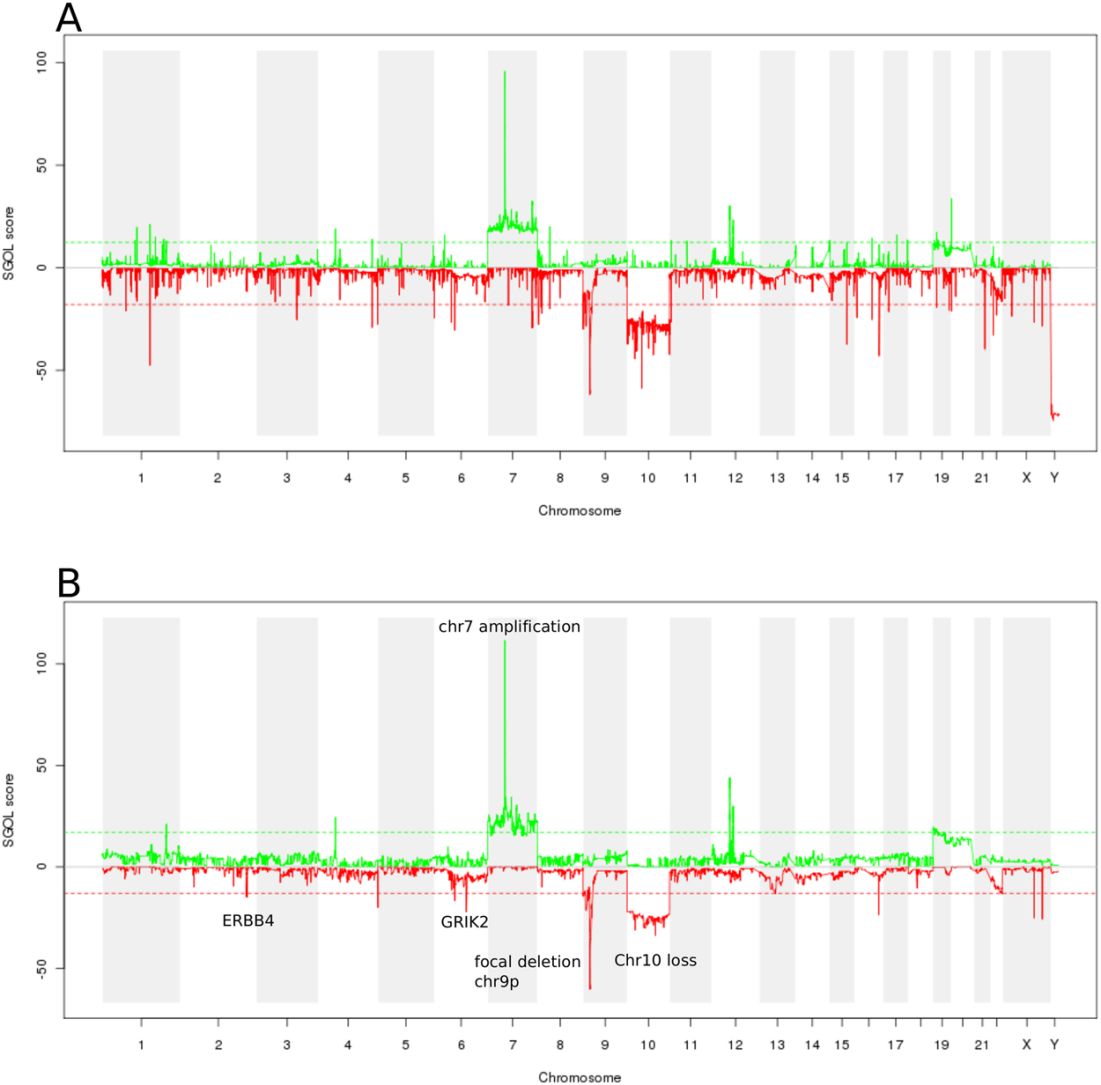
A chgMCR plot of 46 TCGA-GBM samples. (**A**) SNP array data and (**B**) VCF2CNA data are shown.

To further test whether VCF2CNA accurately captures the CNA patterns in samples with library artifacts, we applied the cghMCR algorithm^14^. This package in R Bioconductor provides functions to identify genomic regions of interest based on segmented copy number data from multiple samples. We used this functionality to depict these common gains and losses across all 46 samples from either VCF2CNA profiles or SNP array–derived CNA profiles (downloaded from TCGA). The results are quantified by a segment gain or loss (SGOL) score. Although the signal from VCF2CNA contained less noise than did the signal from the SNP array in most samples (Supplementary File s1), both profiles reveal common recurrently amplified and/or lost regions (Fig. 4). These changes included chromosome-level changes (i.e., chr7 amplifications and loss of chr10) and segmental CNAs (i.e., focal deletion of the *CDKN2A/B* locus on chr9p)^15^. Moreover, VCF2CNA identified recurrent losses in *ERBB4* on chr2q and *GRIK2* on chr6q that were absent in the SNP array profiles. *ERBB4* encodes a transmembrane receptor kinase that is essential for neuronal development^16^. It is frequently mutated in patients with non-small cell lung cancer^17^, and silencing of *ERBB4* through DNA hypermethylation is associated with poor prognosis in primary breast tumors^18^. Similarly, *GRIK2* is a candidate tumor suppressor gene that is frequently deleted in acute lymphocytic leukemia^19^ and silenced by DNA hypermethylation in gastric cancer^20^.

**Figure 5:**
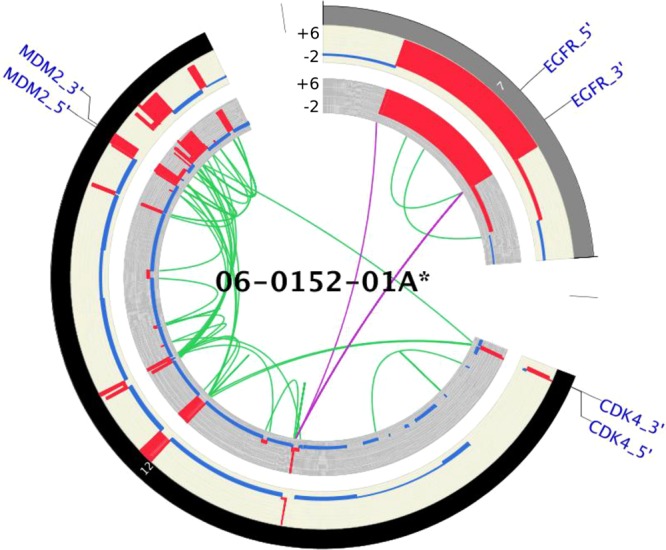
A Circos plot of VCF2CNA (outer ring) and CONSERTING (inner ring), depicting high-amplitude focal CNA segments in TCGA-GBM sample 06-0152-01A. Included in these segments are the known cancer genes *EGFR*, *CDK4*, and *MDM2*. CNA range is specified for each sample.

Amplifications such as double minute chromosomes and homogeneously staining regions represent a common mechanism of oncogene overexpression in tumors^21^. Among the 46 TCGA-GBM samples analyzed, VCF2CNA identified double minute chromosomes in 34 samples affecting the *EGFR*^22^, *MDM2*^23^, *MDM4*^24^*, PDGFRA*^25^*, HGF*^26^*, GLI1*^27^*, CDK4*^28^, and *CDK6*^29^ genes (Fig. 5 and Supplementary File s3). These events consisted of high-level amplifications in 21 samples with potential fractured genome patterns (Supplementary File s3a) and 13 previously reported samples (Supplementary File s3b)^7,30^.

### CNA analysis of TARGET-NBL data

We applied VCF2CNA to the TARGET-NBL dataset^11^ downloaded from dbGap (assession number: phs000467). This dataset consists of 146 tumors with matched normal WGS samples, sequenced with CGI technology. Because the ligation-based CGI technology has notable differences in the detection of single nucleotide variants (SNVs) and insertions/deletions (indels) compared to Illumina systems^31^, this dataset provided an opportunity to evaluate VCF2CNA’s robustness using different sequencing platforms.

**Figure 6:**
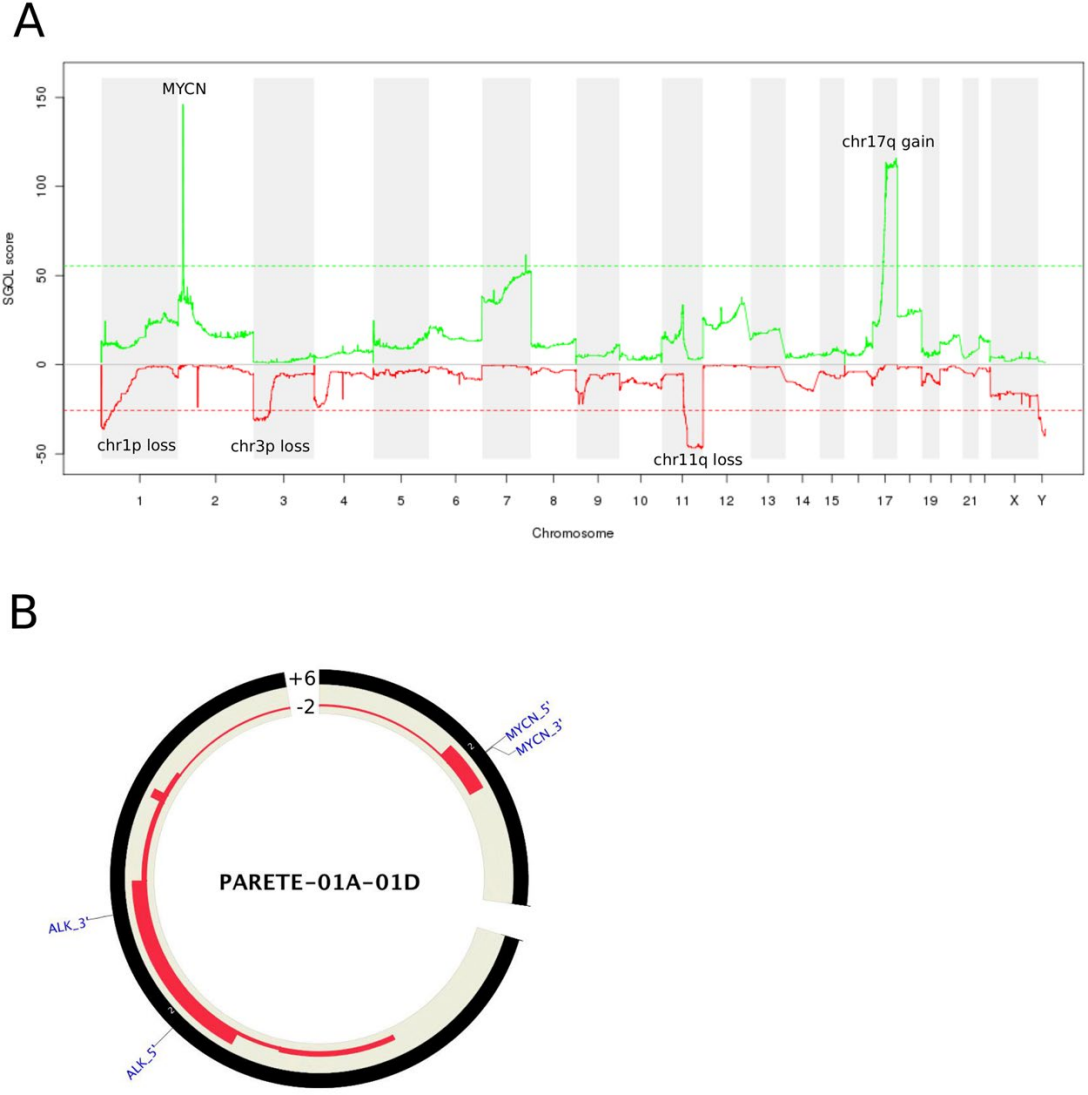
Analysis of the TARGET-NBL dataset, consisting of 146 tumors. (**A**) A chgMCR plot in which green depicts regions of copy-number gain and red depicts regions of copy-number loss. (**B**) A Circos plot showing a focal gain on chromosome 2 for *MYCN* and *ALK5* for sample PARETE-01A-01D. CNA range is specified.

We used VCF2CNA to perform cghMCR analysis with CNA profiles and observed a genome pattern similar to that reported for SNP array platforms (Fig. 6A)^32^. In addition to loss of large regions on chr1p, 3p, and 11q and a broad gain of chr17q, VCF2CNA found frequent focal amplifications of *MYCN* in NBL tumors and several potential cancer-related CNAs, including high-level amplifications of *CDK4* (1 tumor), and *ALK* (2 tumors) (Fig. 6B).

High-level amplification of *MYCN* is a known oncogenic driver found in ~25% of pediatric patients with NBL, and is associated with aggressive tumors and poor prognosis^33^. A subset of 32 tumors in the TARGET-NBL cohort contains clinically validated amplifications of *MYCN*. Although the CGI’s hidden Markov CNA model (unpublished) reported *MYCN* amplifications in 15 of these 32 tumors, VCF2CNA successfully identified high-level amplifications in 31 tumors. In the clinically validated *MYCN*-amplified sample that went undetected by VCF2CNA, a follow-up review revealed that tumor heterogeneity and sampling bias most likely contributed to the discrepancy. Moreover, VCF2CNA predicted two additional *MYCN* amplification events among the remaining tumor samples, indicating that VCF2CNA can identify clinically relevant CNAs that were undetected by traditional methods of CNA detection. The high-level concordance with clinically validated data provides a strong indication that VCF2CNA is applicable to multiple tumor types collected from different sequencing platforms.

### CNA analysis of rhabdomyosarcoma data to compare WXS and WGS

**Figure 7:**
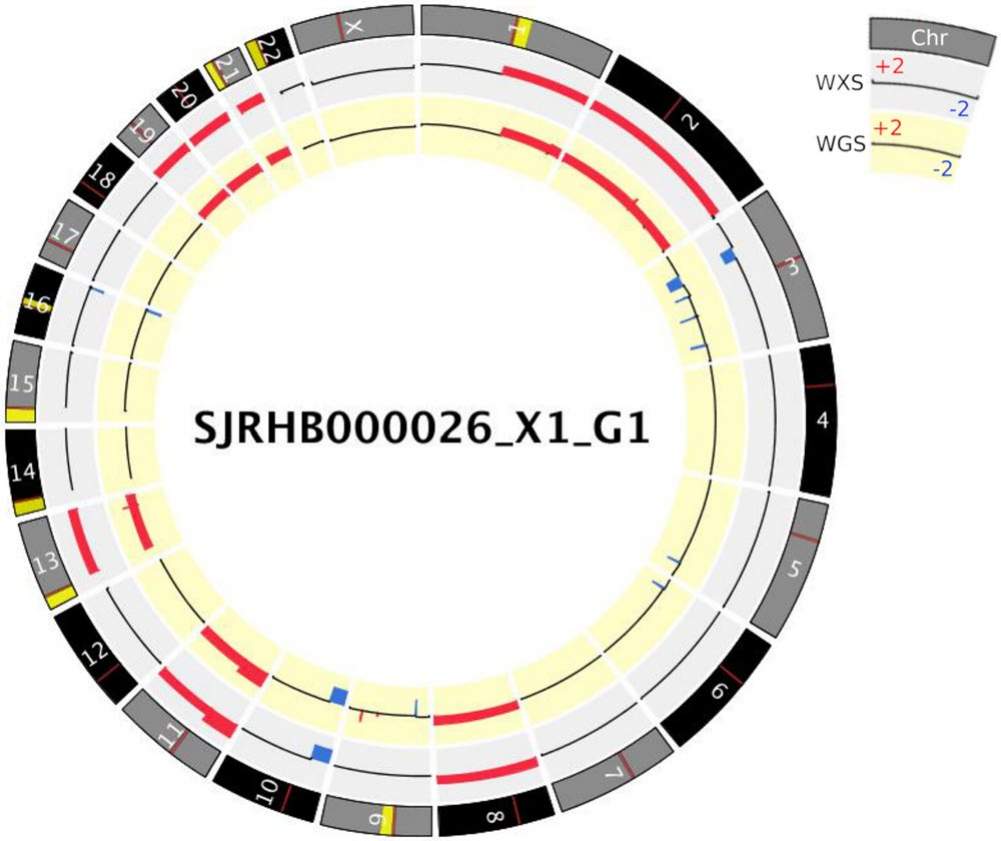
Somatic CNAs computed using VCF2CNA for paired whole-exome and whole-genome Rhabdomyosarcoma xenograph sample SJRHB000026_X1_G1.

Although, WGS provides unbiased coverage measurements across the genome, whole exome sequencing (WXS) offers characterization of the coding regions of the genome (2% of genome) at much higher depth, which provides a convenient and inexpensive alternative to WGS and has been widely adopted in large scale genome profiling projects and clinical settings. Due to major design differences between the two platforms, we evaluated the consistency of copy number alteration detection between whole exome and whole genome sequencing, using a set of rhabdomyosarcoma samples that were sequenced on both platforms^12^. We observed highly consistent CNA profiles between WGS and WXS platforms (mean F1 score 0.97 on a set of 15 rhabdomyosarcoma xenograph samples). While focal changes are more likely to be missed in the WXS platform compared to the WGS platform, VCF2CNA reliably detects large CNAs from both WGS and WXS platforms (Fig. 7, Supplementary File s5).

### CNA-based purity estimation

Using the absolute copy number result for each segment identified through VCF2CNA, and B-allele frequencies (BAFs) computed from the paired tumor-normal VCF file, we developed an algorithm to estimate tumor purity using segments with a single copy number gain or loss in VCF2CNA. Briefly, for germline heterogeneous single nucleotide polymorphisms (SNPs, base BAF of 0.5), the extent of loss of heterozygosity (LOH) can be measured by the absolute difference between the B-allele fraction in tumor and that in germline sample. LOH is the result of copy number alteration and/or copy neutral-LOH in tumor cells. We used LOH signals in copy neutral or single-copy gain/loss regions (between single-copy chromosome loss and single-copy chromosome gain) to estimate tumor purity.

Using purity estimates from various regions within the genome we performed an unsupervised clustering analysis using the mclust package (version 5.4) in R (version 3.4.0). The tumor purity of the sample was defined as the highest cluster center value among all clusters. We estimated Tumor purity for 15 matched tumor-normal xenograph Rhabdomyosarcoma WGS samples. (Table 3). All but one case had a tumor purity prediction near 100%, consistant with the notion that the most mouse-derived reads won’t be mapped to the human genome assembly^34,35^. The sample SJRHB010468_X1_G1 showed extensive subclonal CNAs across multiple chromosomes (Supplementary File s5). While subclonal CNAs are not indicative of low purity, the extensive subclonal copy number segments result in an incorrect tumor purity estimation (0.533), which is a limitation of the algorithm. The Mutant allele fraction (MAF) density plot for somatic single nucleotide variations (SNVs) detected in diploid regions, revealed a subclone in 50% of the tumor cells, which harbors more than 75% of the detected SNVs (Supplementary File s6).

**Table 3:** Purity estimation of Rhabdomyosarcoma paired tumor-normal WGS samples.

Sample	Purity Estimation	Diploid Region
SJRHB011_X_G	0.993	Chr 22
SJRHB011_Y_G	0.997	Chr 21
SJRHB012_Y_G	0.996	Chr 18
SJRHB013_X_G	0.995	Chr 21
SJRHB000026_X1_G1	0.999	Chr 4
SJRHB000026_X2_G1	0.999	Chr 4
SJRHB010463_X16_G1	1.000	Chr 21
SJRHB010468_X1_G1	0.533	Chr 20
SJRHB010927_X1_G1	1.000	Chr 18
SJRHB010928_X1_G1	0.996	Chr 7
SJRHB012405_X1_G1	0.993	Chr 22
SJRHB013757_X2_G1	0.995	Chr 17
SJRHB013758_X1_G1	0.999	Chr 18
SJRHB013758_X2_G1	0.996	Chr 6
SJRHB013759_X1_G1	0.998	Chr 13 19020701-26516137

## Discussion and Conclusions

We developed VCF2CNA for the systematic and robust detection of CNAs from VCF and other genotyping variant call formats, which can be derived from various sequencing platforms and/or alignment file formats (e.g. BAM, CRAM, Petagene, etc.). Analysis of 192 paired tumor–normal WGS samples sequenced on multiple platforms demonstrates that VCF2CNA is robust to library construction artifacts and captures medium to large CNA segments with high accuracy. The analysis in tumor samples sequenced on both WGS and WXS platforms further suggests that VCF2CNA produces highly consistent CNA profiles from both platforms. VCF2CNA identified recurrent losses in potential tumor suppressors that were undetectable by alternative approaches. The algorithm attempts to automatically determine the diploid region in tumor samples and uses that as the reference. However, in samples with genome-wide duplication, it will not be detected by VF2CNA or any read depth-based algorithm. To address this, VCF2CNA allows the user to define a reference chromosome/region.

VCF2CNA was designed with SNPs that were (on average) thousands of base pairs apart, which limits support for identifying focal copy-number changes. Therefore, state-of-the-art CNA algorithms have superior detection power for focal low-amplitude CNAs in high-quality, high-coverage WGS data.

VCF2CNA includes a method to estimate overall tumor purity for samples with sufficient number of purity estimates for segments containing single copy number gain or loss. Most WXS samples contained too few purity estimates to produce a reliable purity estimation, (a minimum of 20 segments required). The tumor purity estimation in VCF2CNA is derived from CNAs and LOH signals and therefore, the result will be biased if tumor cells do not have these leisions or these leisions are primarily identified in a subclone. The final tumor purity estimation should be compiled from various analyses, including CNA, SNV, and pathology-based evaluation, etc.

In conclusion, VCF2CNA is a web-based tool (with an option of local installation) that is capable of accurate and efficient detection of CNAs and tumor purity from variants called from high-coverage WGS and WXS data sequenced on various platforms.

## Methods

### Server availability

The webserver for VCF2CNA is available at https://vcf2cna.stjude.org. The downloadable executable is available at http://www.github.com/XCLab/VCF2CNA.

### Parameter definitions

The Specify Diploid Chromosome parameter normalizes results by the specified chromosome. The Median Normal Coverage parameter permits input of the median coverage value of SNPs from normal samples. The Minimum Scale Factor (autosomes) parameter is multiplied by the median to compute the minimum coverage value. The Maximum Scale Factor (autosomes) parameter is multiplied by the median to compute the maximum coverage value. The Minimum X Scale Factor is the minimum scale factor for chromosome X. The Maximum X Scale Factor is the maximum scale factor for chromosome X. The Sample Order (VCF format only) parameter defines the ordering of tumor and normal samples. VCF inputs must include tumor and normal data after the FORMAT field. Selecting the Tumor/Normal button assigns the tumor data to the first field after FORMAT and normal data to the second field. The Normal/Tumor radio button specifies the reverse order.

### Input data for VCF2CNA

The input for VCF2CNA analysis includes VCF, MAF, and the variant file format produced by the Bambino program. A fixed window size of 100 bp is used to obtain the mean coverage for each window. Windows with no variants are ignored. The mean read depth per window can be normalized to a set of reference diploid chromosomal regions by using the same criteria as CONSERTING or specified via the Specify Diploid Chromosome parameter.

### Tumor purity estimation

Basic Definitions:

1. B Allele Fraction (BAF): the frequency a given base does not match the corresponding reference sequence, divided by the read depth at that position.
2. Loss of Heterozygosity (LOH): the absolute value of the difference between the BAF of the tumor sample and the BAF of the germline sample at heterozygous sites. This is also referred to as allelic imbalance (AI).
3. Copy Number Alteration (CNA): Inferred copy number change in the tumor sample, where +1/0/−1 represents one copy gain/no change/one copy loss, respectively.

Key Relationships:

1. BAF cluster 1: The location of the left cluster center
  1 $$\begin{array}{c}{\rm{Left}}\,{\rm{Center}}=\frac{1+{\rm{CNA}}}{2+{\rm{CNA}}}\end{array}$$
2. BAF cluster 2: The location of the right cluster center
  2 $$\begin{array}{c}{\rm{Right}}\,{\rm{Center}}=\frac{1}{2+{\rm{CNA}}}\end{array}$$
3. BAF separation: The distance between the cluster centers of two populations of BAFs is given by 2(LOH). This is also given by the absolute value of the difference between BAF cluster 1 and 2.

3 $$\begin{array}{c}{\rm{BAF}}\,{\rm{Distance}}=|\frac{1+{\rm{CNA}}}{2+{\rm{CNA}}}-\frac{1}{2+{\rm{CNA}}}|=|\frac{{\rm{CNA}}}{2+{\rm{CNA}}}|=2({\rm{LOH}})\end{array}$$

### VCF2CNA output

The VCF2CNA pipeline produces an output text file including the following fields:

1. Seg.mean: A value of 1.0 corresponds to 2 copy gain, 0.5 corresponds to 1 copy gain, 0 corresponds to no gain or loss, −0.5 to 1 copy loss and −1.0 to 2 copy loss.
2. Gmean: A value of 2.0 corresponds to a diploid sample, 0.5 corresponds to 1 copy loss and 0 corresponds to 2 copy loss.

### BAF calculation

The distribution of BAF values is related to the underlying copy number changes. They represent the total number of reads matching one of two allele types at a given heterozygous site. The A allele is the allele matching the germline genome, while the B allele is the corresponding unmatched allele. At a heterozygous site, the expected BAF value in the germline sample is 0.5. Copy number changes at these heterozygous sites in tumor samples may cause a deviation from 0.5. This LOH depends on the absolute copy number changes. Analysis of BAF plots of heterozygous sites for copy number change regions depict uni/bimodal distribution BAFS. The distance between BAF distributions varies due to varying combinations of tumor purity and copy number changes. Using absolute copy number changes computed from VCF2CNA and LOH measurements from heterozygous BAF sites we compute tumor purity.

To accomplish this we combine the segmented CNA output from VCF2CNA with BAF values from heterozygous sites in germline samples of paired tumor-germline samples. Each segment has the same CNA value. The j-th segment of the tumor genome is given by C_j_ with j = 1,2, …, J. Heterozygous sites in the corresponding normal genome are mapped to these segments using the starting and ending location of the segment. We specify (i,j) to index the i-th heterozygous site on segment j with i = 1, 2, …, I_j_, where I_j_ is the total number of heterozygous sites in segment j. Only BAF sites that fall inside a given VCF2CNA segment are used in analysis.

### Purity derivation

We assume the following populations:

1. x: fraction of cells with a single-copy CNA. (gain x > 0, loss x < 0)
2. y: fraction of cells with CN-LOH. (The chromosome lost is the chromosome lost in CN-LOH, or the chromosome gained is the chromosome gained in CN-LOH).

4 $$\begin{array}{c}{\rm{Measured}}\,{\rm{CNA}}=x\end{array}$$

5 $$\begin{array}{c}{\rm{Measured}}\,{{\rm{LOH}}}_{{\rm{loss}}}=(\frac{1+y}{2+x})-0.5\end{array}$$

6 $$\begin{array}{c}{\rm{Measured}}\,{{\rm{LOH}}}_{{\rm{gain}}}=(\frac{1+x+y}{2+x})-0.5\end{array}$$

7 $$\begin{array}{c}{\rm{Measured}}\,{{\rm{LOH}}}_{{\rm{combined}}}=(\frac{2y+|x|}{4+2x})\end{array}$$

Solve equation 7 for y:

8 $$\begin{array}{c}{\rm{y}}={\rm{LOH}}(2+{\rm{x}})-0.5|{\rm{x}}|\end{array}$$

9 $$\begin{array}{c}{\rm{purity}}=|{\rm{x}}|+{\rm{y}}={\rm{LOH}}(2+{\rm{CNA}})+\frac{|{\rm{CNA}}|}{2}\end{array}$$

### Run-time analysis

Single VCF files must be converted to a paired tumor/normal file before uploading. Alternatively, VCF2CNA accepts MAF and Bambino variant file formats. After uploading files to the server, the median running time was 23 minutes on an intel Xeon E5-2680 processor at 2.70 Ghz with 64 GB RAM. Server processing occurs in two principal steps: (1) preprocessing and SNP information extraction from input files and (2) running the recursive partitioning segmentation.

### F_1_ scoring metric and segmental corroboration

A genomic position was assigned a corroborated CNA call if its computed CNA type (gain or loss) by VCF2CNA matched the call computed by CONSERTING. A CNA segment in the CONSERTING profile was corroborated in the VCF2CNA profile if ≥90% of the segment positions were corroborated. The F_1_ score is given by ${F}_{1}=\frac{2(precision)\,(recall)}{precision+recall}$ . It was used to summarize the accuracy of VCF2CNA, compared with that of CONSERTING.

### VCF2CNA web server pipeline

#### Step1 (snvcounts)

Single nucleotide variant frequencies are computed from the input file. For each chromosome and position, the values computed are TumorMutant, TumorTotal, NormalMutant, and NormalTotal. Additionally, the mean normal coverage is computed.

#### Step2 (consprep)

Using a collection of 625 WGS samples in the Pediatric Cancer Genome Project^36^, we generated an in-house blacklist of suspicious SNPs, many of which are potentially mapping artifacts in the blacklisted regions of ENCODE (https://personal.broadinstitute.org/anshul/projects/encode/rawdata/blacklists/hg19-blacklist-README.pdf). The consprep program reads the SNV count data and incorporates this list to identify heterozygous sites. It also reads a file specifying the number of 100-bp windows in each chromosome. If the total number of reads from the normal sample falls outside of the ranges specified by the options (median, minfactor, maxfactor, xminfactor, or xmaxfactor), the input position is ignored by the consprep step in the pipeline. The –xminfactor and –xmaxfactor settings apply to positions in chrX; the –minfactor and –maxfactor settings apply to all other chromosomes. The minimum coverage is the median multiplied by the –minfactor, and the maximum coverage is the median multiplied by the –maxfactor.

#### Application

To run VCF2CNA, users should navigate to the application home page and click “run application.” The application runs on Google Chrome, Safari, Mozilla Firefox, and Microsoft Internet Explorer 11. Users must provide a valid email in the email address text field. Users will select whether results will be sent to the provided email address as either an email attachment or a link to the result files stored on the server. Once the analysis is complete, the original input file is deleted from the server immediately. If an error occurs during analysis, the input file is stored on the server for 7 days and then purged from the system. The results of the analysis are stored on the server for 14 days. After that time-period, they are deleted from the server.

Default run parameters may be modified depending on job specifications. Users should select the input file and click the “upload/run” button. The browser window should not be killed during the file upload. Once the file has been successfully uploaded, a notification will be displayed in the browser window and the user may discard the window.

### Rationale for not using the reciprocal-overlap rule

To compare CNA calls from different algorithms, the reciprocal 50% overlap criterion^31^ is commonly used. This rule is not suitable when two CNA calls are derived from platforms with different powers in detecting focal CNAs. A considerably larger average distance occurred between adjacent probes. VCF2CNA-derived CNA calls have an inherently lower resolution than does CONSERTING. When a focal CNA identified through CONSERTING occurs on top of a large CNA fragment, CONSERTING breaks the region into multiple segments. Although the CNA fragments in the region are largely corroborated between the two CNA callers, potentially none of these fragments satisfied the rule of reciprocal 50% overlap (Supplementary File S4).

## Supplementary information

Supplementary material

## Data Availability

WGS/WXS datasets used in the study were downloaded from dbGaP (https://dbgap.ncbi.nlm.nih.gov). The TCGA-GBM data were downloaded from dbGaP (Accession Number: phs000178.v8.p7) and included 46 samples. The TARGET-NBL data were downloaded from dbGap (Accession Number: phs000467) and included 146 samples. RMS genomic data files have been deposited in the European Bioinformatics Institute (EMBL-EBI) under Accession Number EGAS00001002528. VCF2CNA is available at https://vcf2cna.stjude.org and http://www.github.com/XCLab/VCF2CNA.
