# Supplementary material for: VCF2CNA: A tool for efficiently detecting copy-number alterations in VCF genotype data and tumor purity
Source: Sci Rep. 2019 Jul 17;9:10357. doi: 10.1038/s41598-019-45938-x (PMC6637131; doi:10.1038/s41598-019-45938-x)

Supplementary material for VCF2CNA: A tool for efficiently detecting copy-number alterations in VCF genotype data and tumor purity

Daniel K. Putnam, Xiaotu Ma, Stephen V. Rice, Yu Liu, Scott Newman, Jinghui Zhang and Xiang Chen*

Department of Computational Biology, St Jude Children's Research Hospital, Memphis TN, USA

*Corresponding author

E-mail: xiang.chen@stjude.org

S1: Panel A

Circos plot of CONSERTING (outer ring), VCF2CNA (middle ring), and SNP array (inner ring) for 24 TGA-GBM samples with a fractured gene signature. Legend depicts CNA range of data

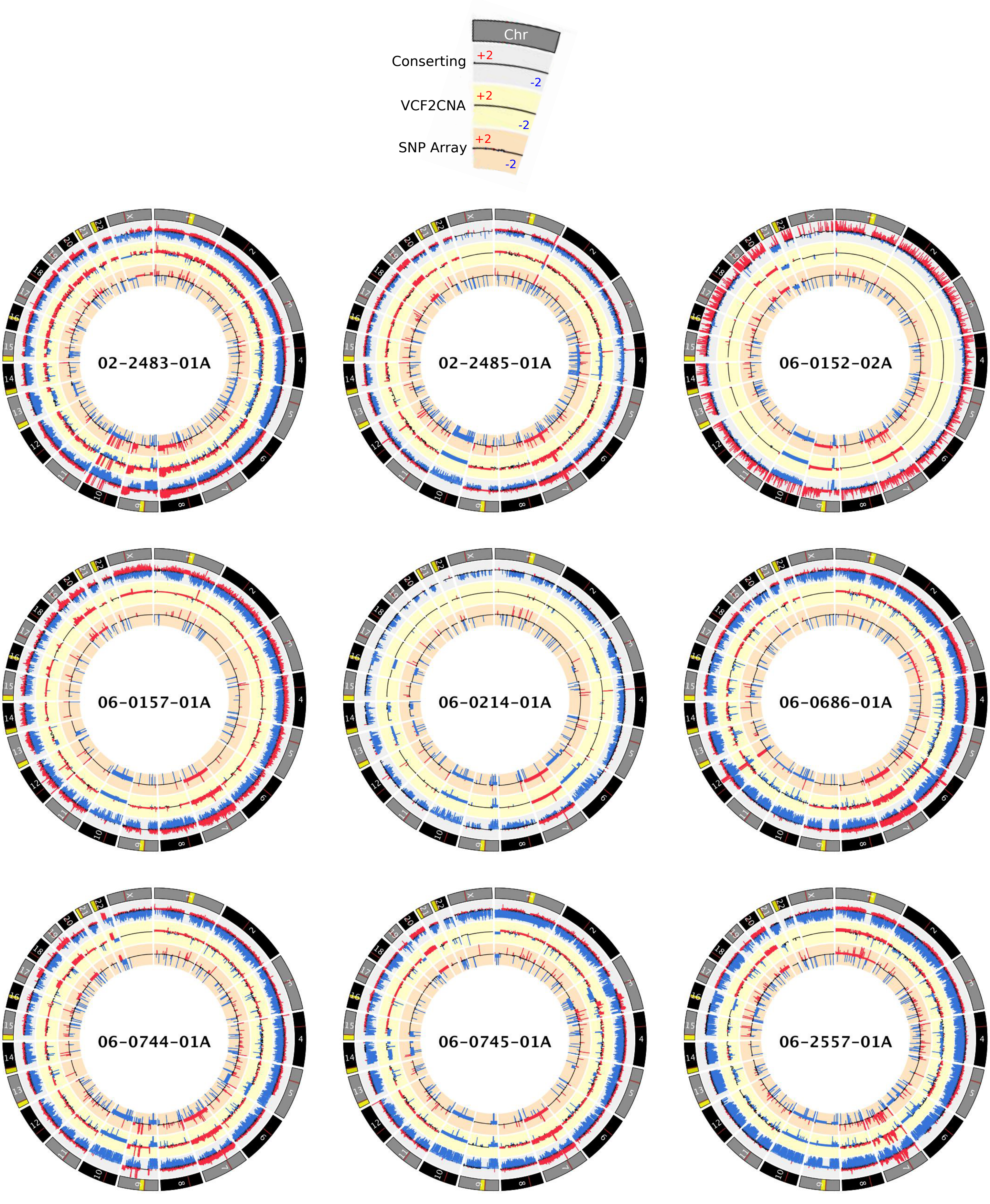

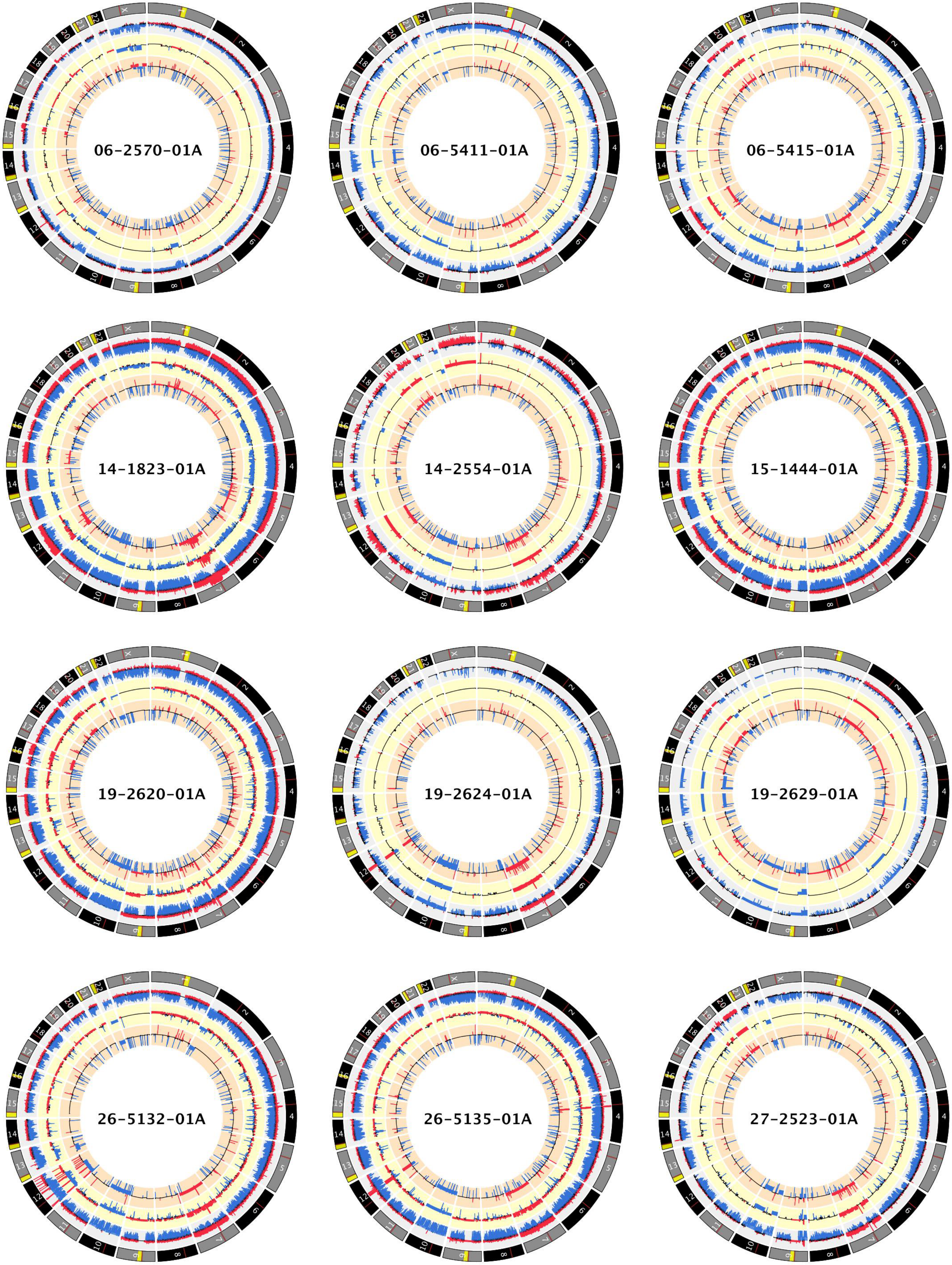
06-2570-01A
06-5411-01A
06-5415-01A
14-1823-01A
14-2554-01A
15-1444-01A
19-2620-01A
19-2624-01A
19-2629-01A
26-5132-01A
26-5135-01A
27-2523-01A

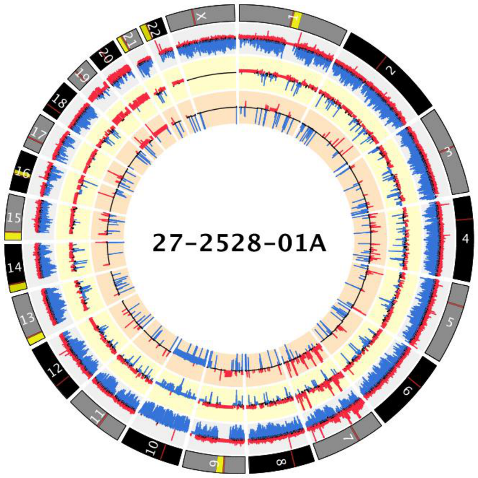
27-2528-01A

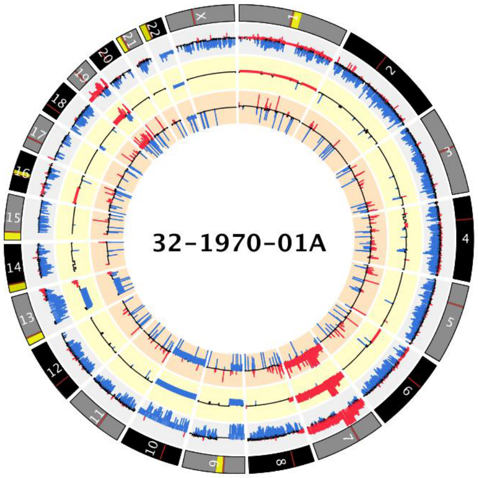
32-1970-01A

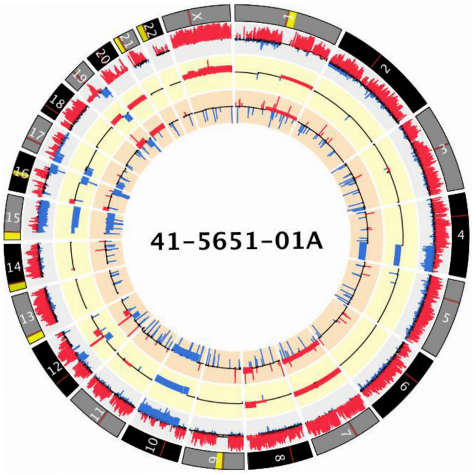
41-5651-01A

S1: Panel B

IGV plot of CONSERTING (CON), VCF2CNA (V2C) and SNP array (SNP) for 24 TGA-GBM samples with a fractured gene signature.

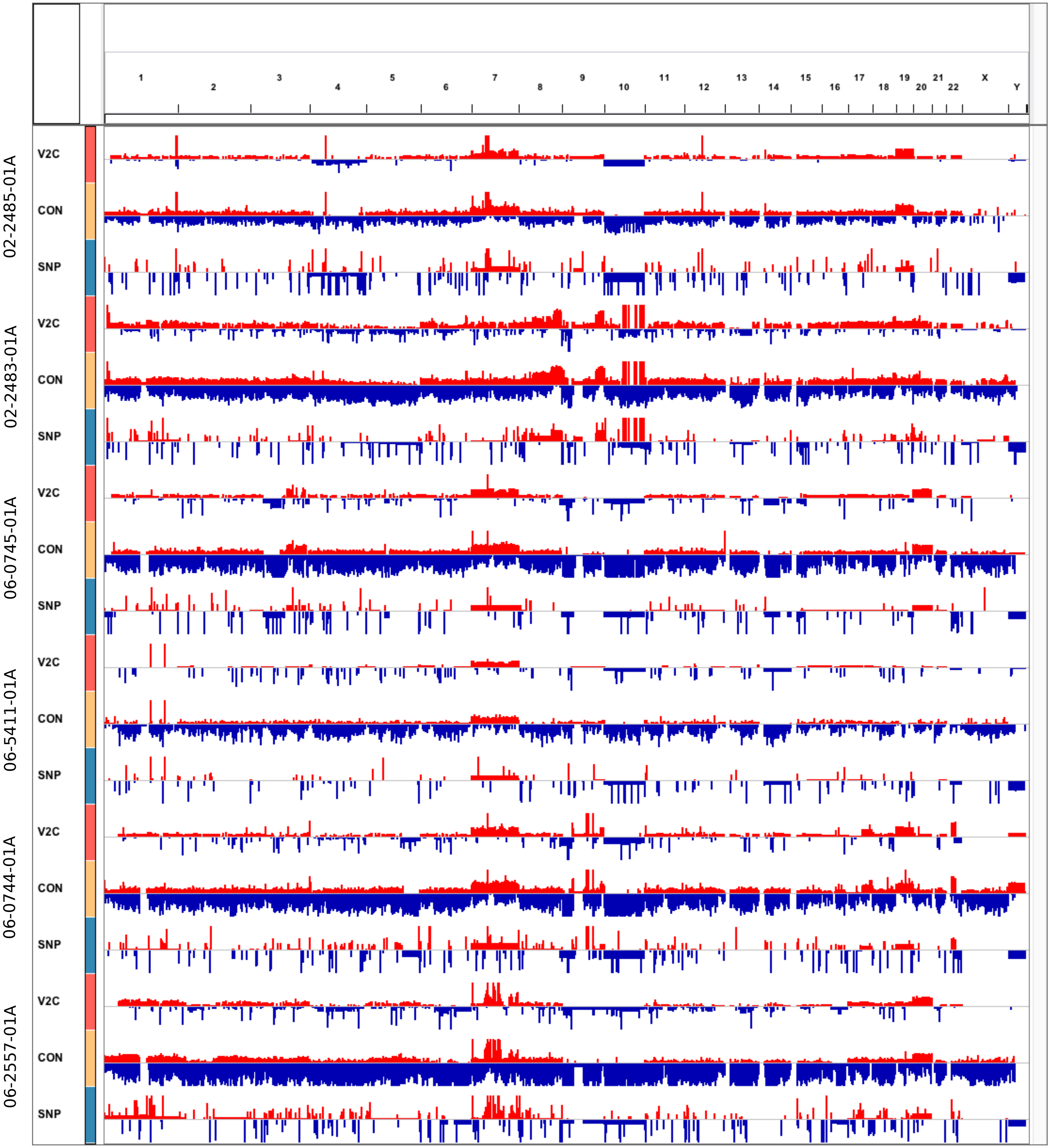

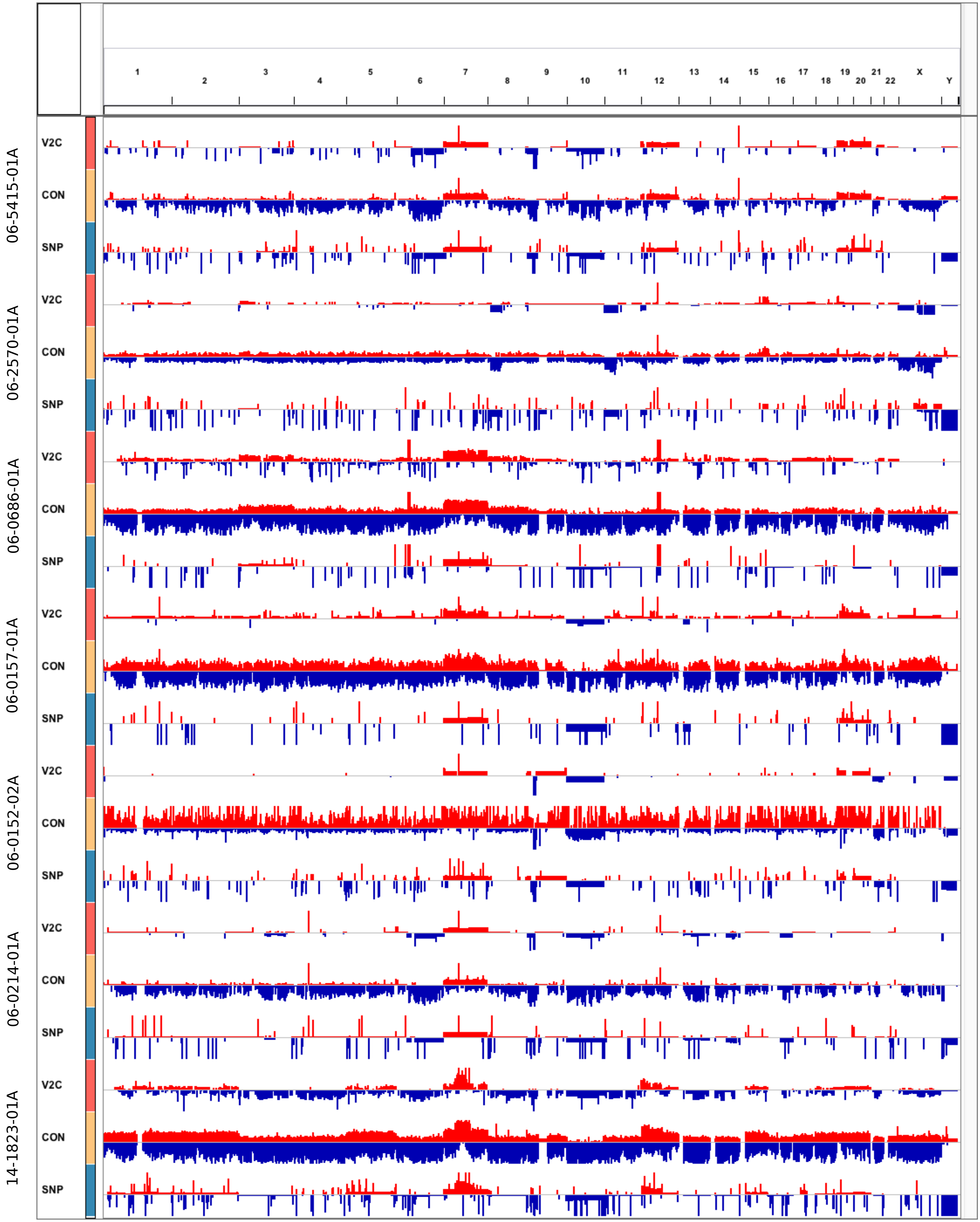
1
2
3
4
5
6
7
8
9
10
11
12
13
14
15
16
17
18
19
20
21
22
X
Y
06-5415-01A
V2C
CON
SNP
06-2570-01A
V2C
CON
SNP
06-0686-01A
V2C
CON
SNP
06-0157-01A
V2C
CON
SNP
06-0152-02A
V2C
CON
SNP
06-0214-01A
V2C
CON
SNP
14-1823-01A
V2C
CON
SNP

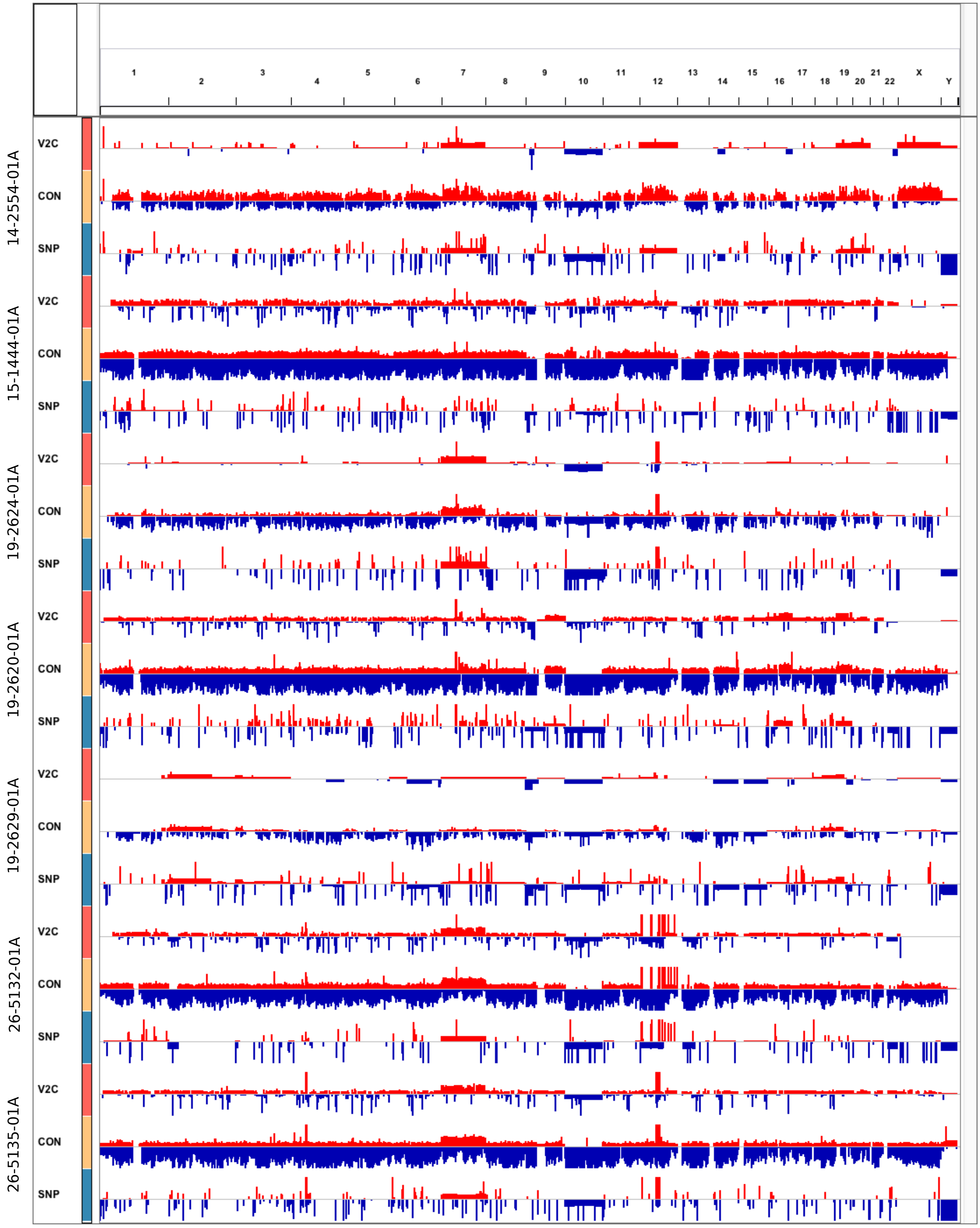
1
2
3
4
5
6
7
8
9
10
11
12
13
14
15
16
17
18
19
20
21
22
X
Y
14-2554-01A
15-1444-01A
19-2624-01A
19-2620-01A
19-2629-01A
26-5132-01A
26-5135-01A
V2C
CON
SNP

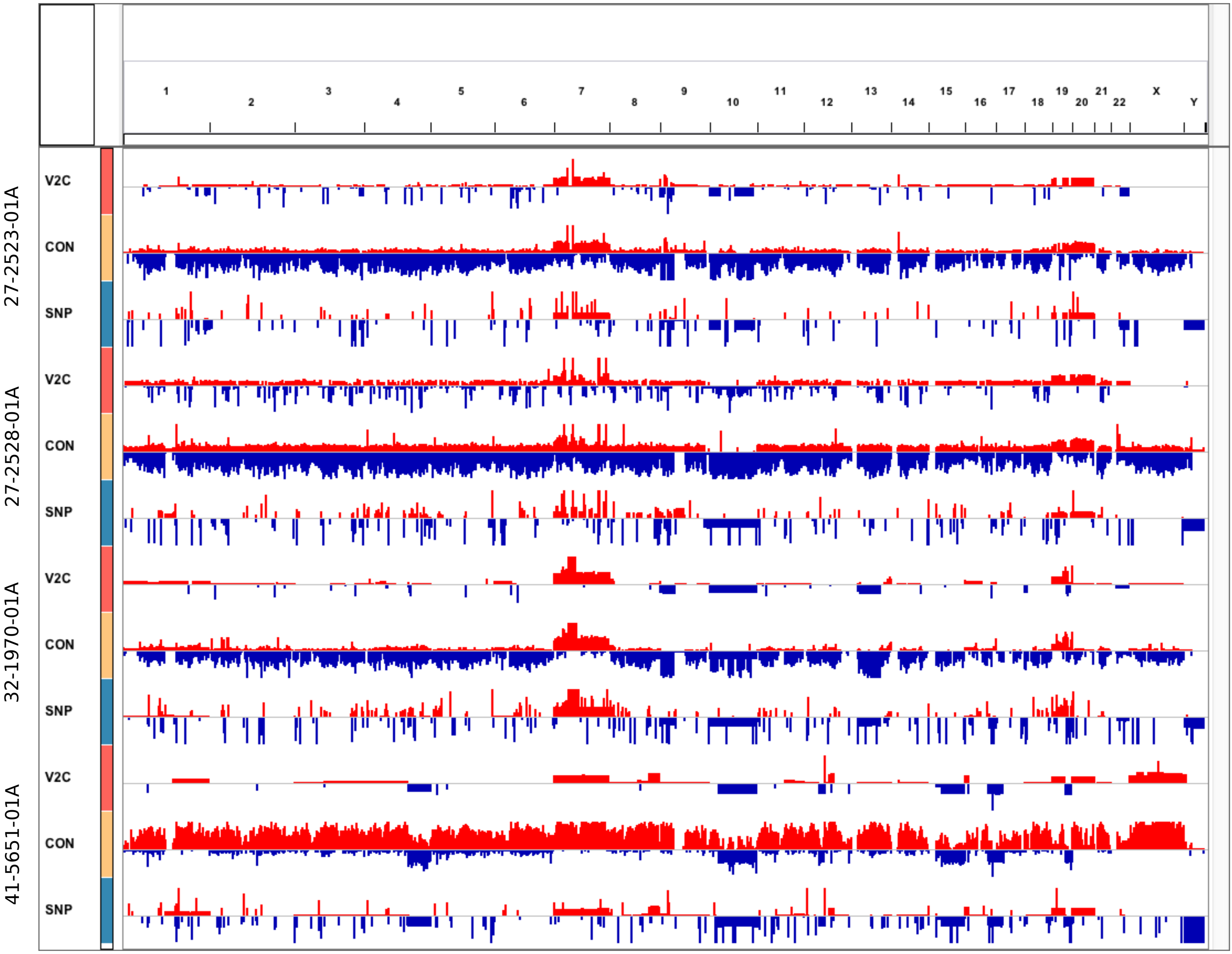

1
2
3
4
5
6
7
8
9
10
11
12
13
14
15
16
17
18
19
20
21
22
X
Y
27-2523-01A
27-2528-01A
32-1970-01A
41-5651-01A
V2C
CON
SNP

S2: Panel A

Circos plot of CONSERTING (outer ring) and VCF2CNA (inner ring) for all 22 TCGA-GBM saples wihtout a fractured gene signature. Legend depicts CNA range of data.

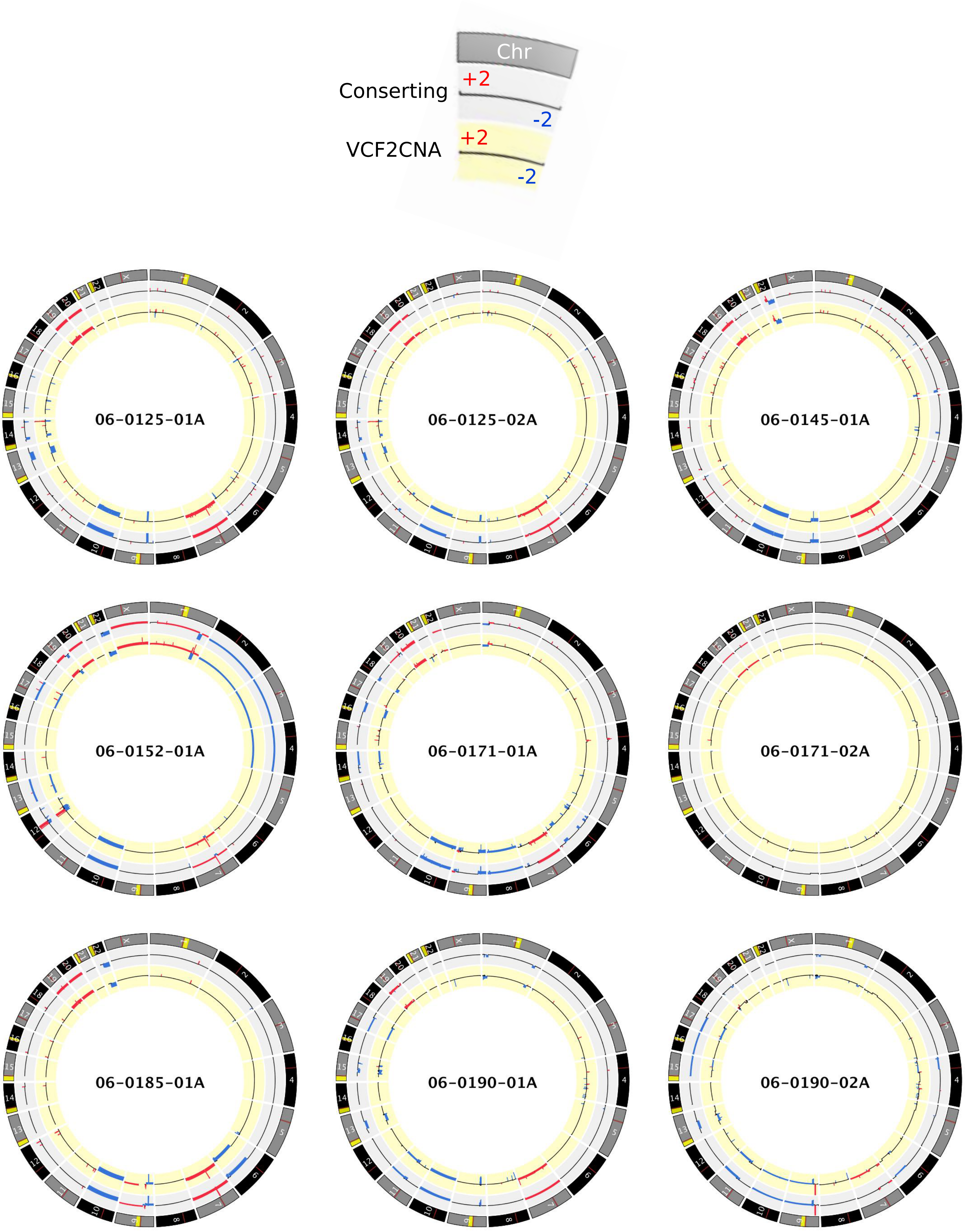

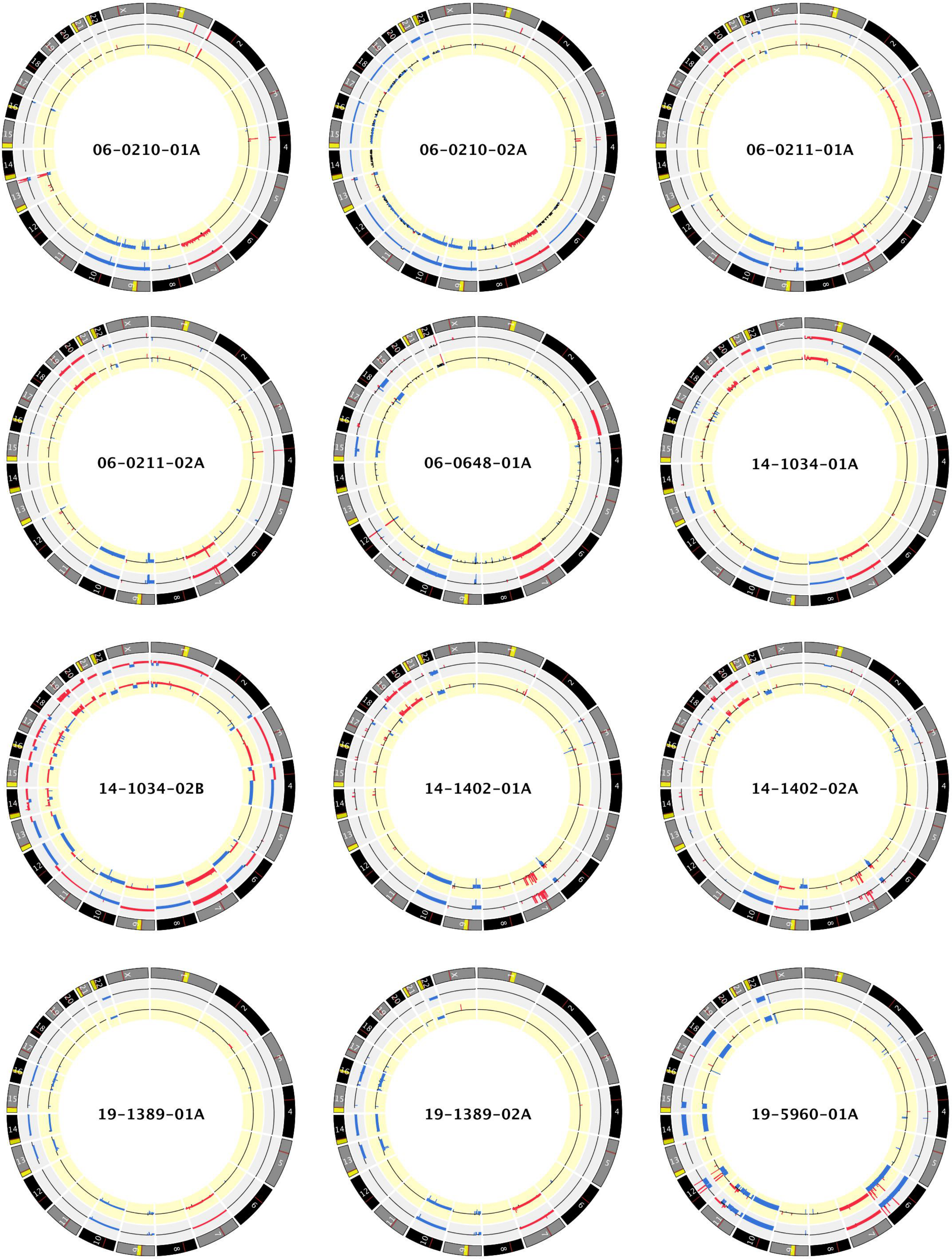
06-0210-01A
06-0210-02A
06-0211-01A
06-0211-02A
06-0648-01A
14-1034-01A
14-1034-02B
14-1402-01A
14-1402-02A
19-1389-01A
19-1389-02A
19-5960-01A

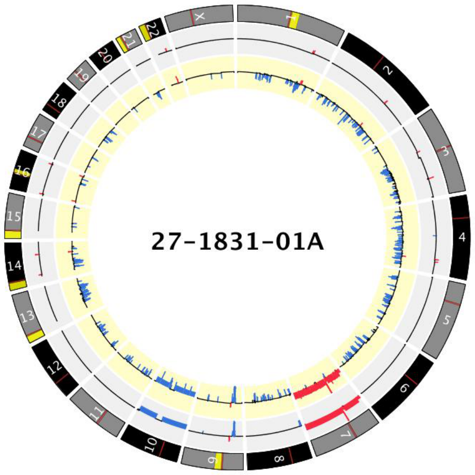
27-1831-01A
1
2
3
4
5
6
7
8
9
10
11
12
13
14
15
16
17
18
19
20
21
22
X

S2: Panel B

IGV plot of CONSERTING (CON) and VCF2CNA(V2C) for all 22 TCGA-GBM samples without a fractured gene signature

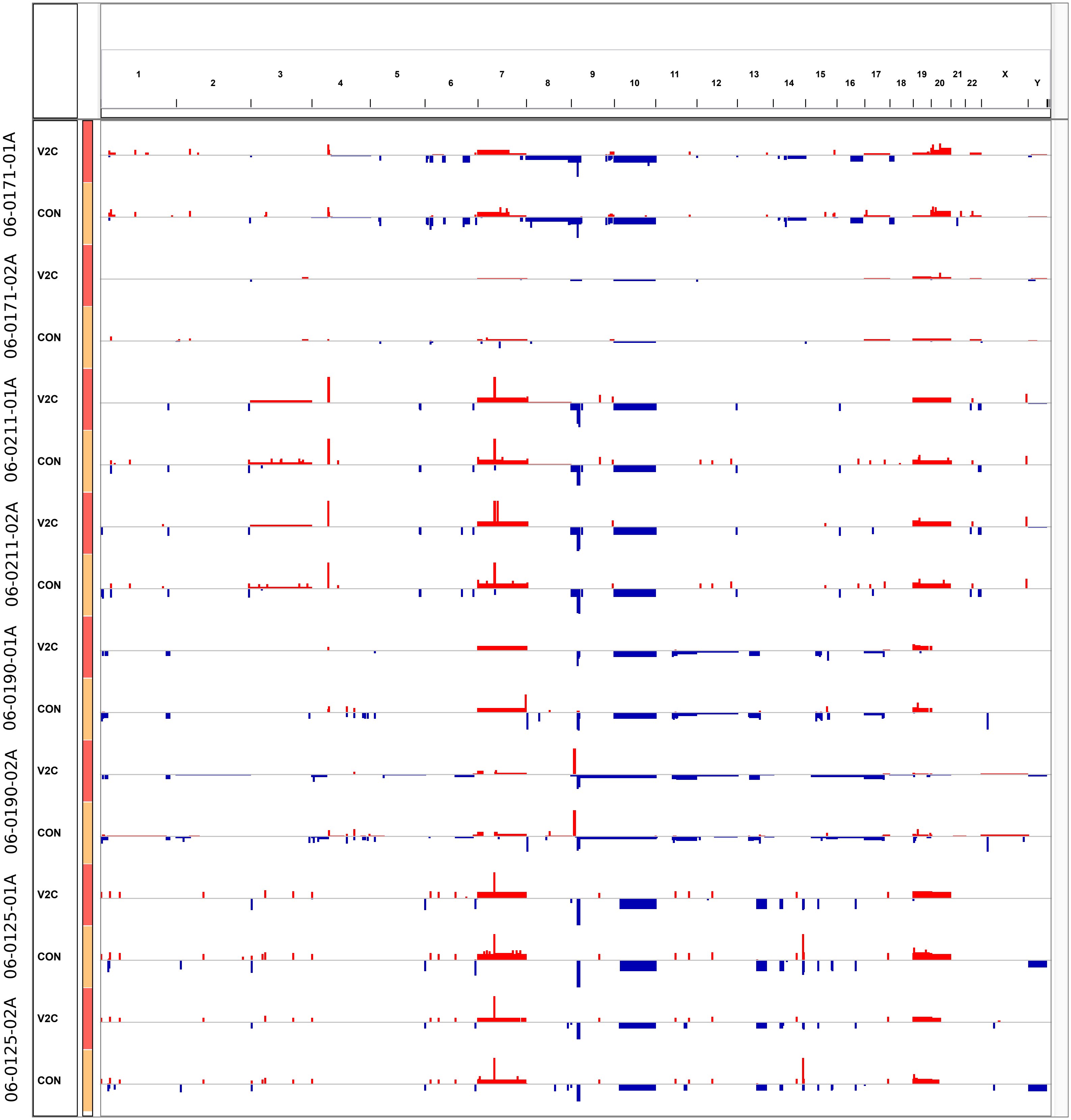

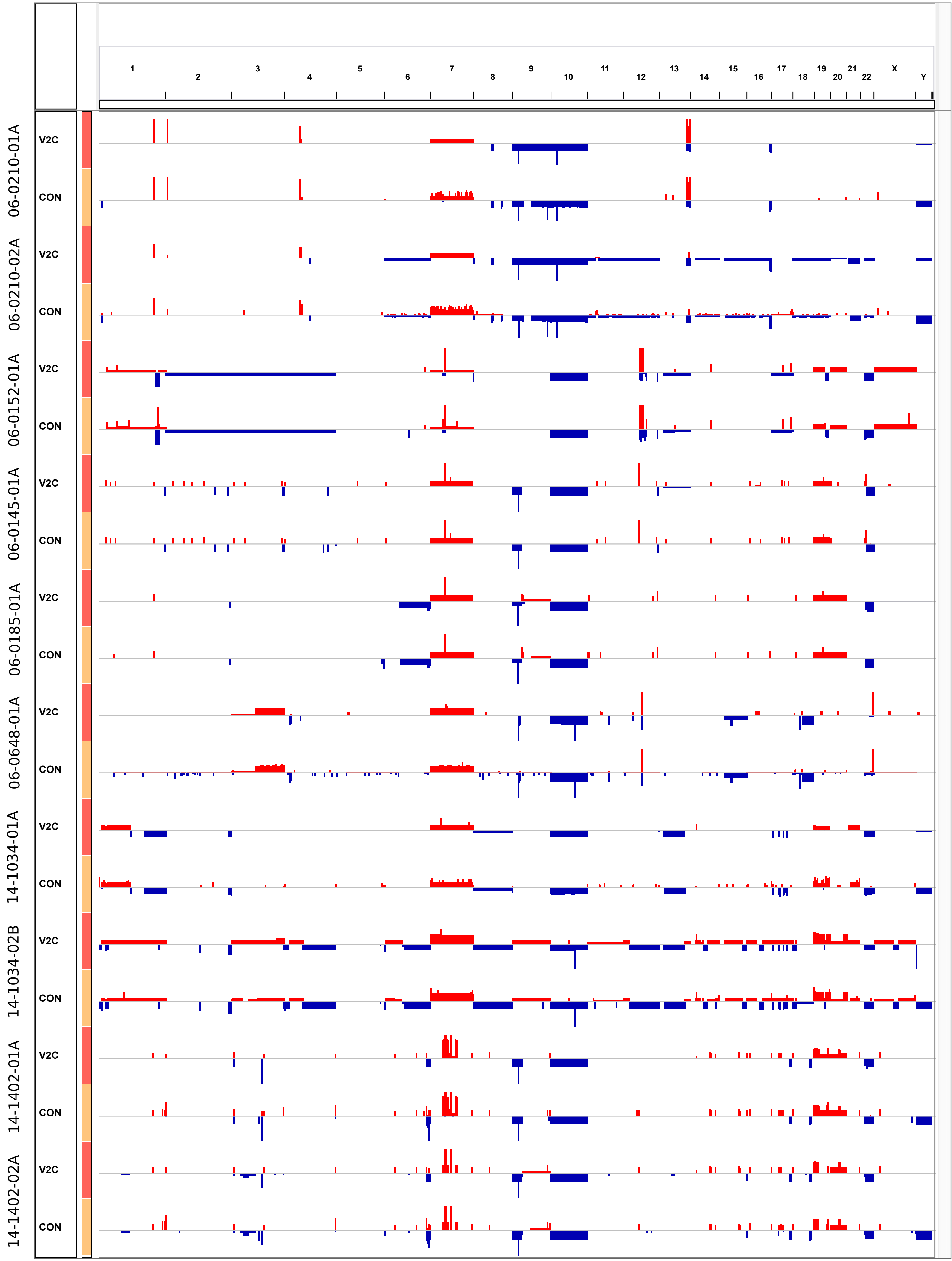

1
2
3
4
5
6
7
8
9
10
11
12
13
14
15
16
17
18
19
20
21
22
X
Y
06-0210-01A
V2C
CON
06-0210-02A
V2C
CON
06-0152-01A
V2C
CON
06-0145-01A
V2C
CON
06-0185-01A
V2C
CON
06-0648-01A
V2C
CON
14-1034-01A
V2C
CON
14-1034-02B
V2C
CON
14-1402-01A
V2C
CON
14-1402-02A
V2C
CON

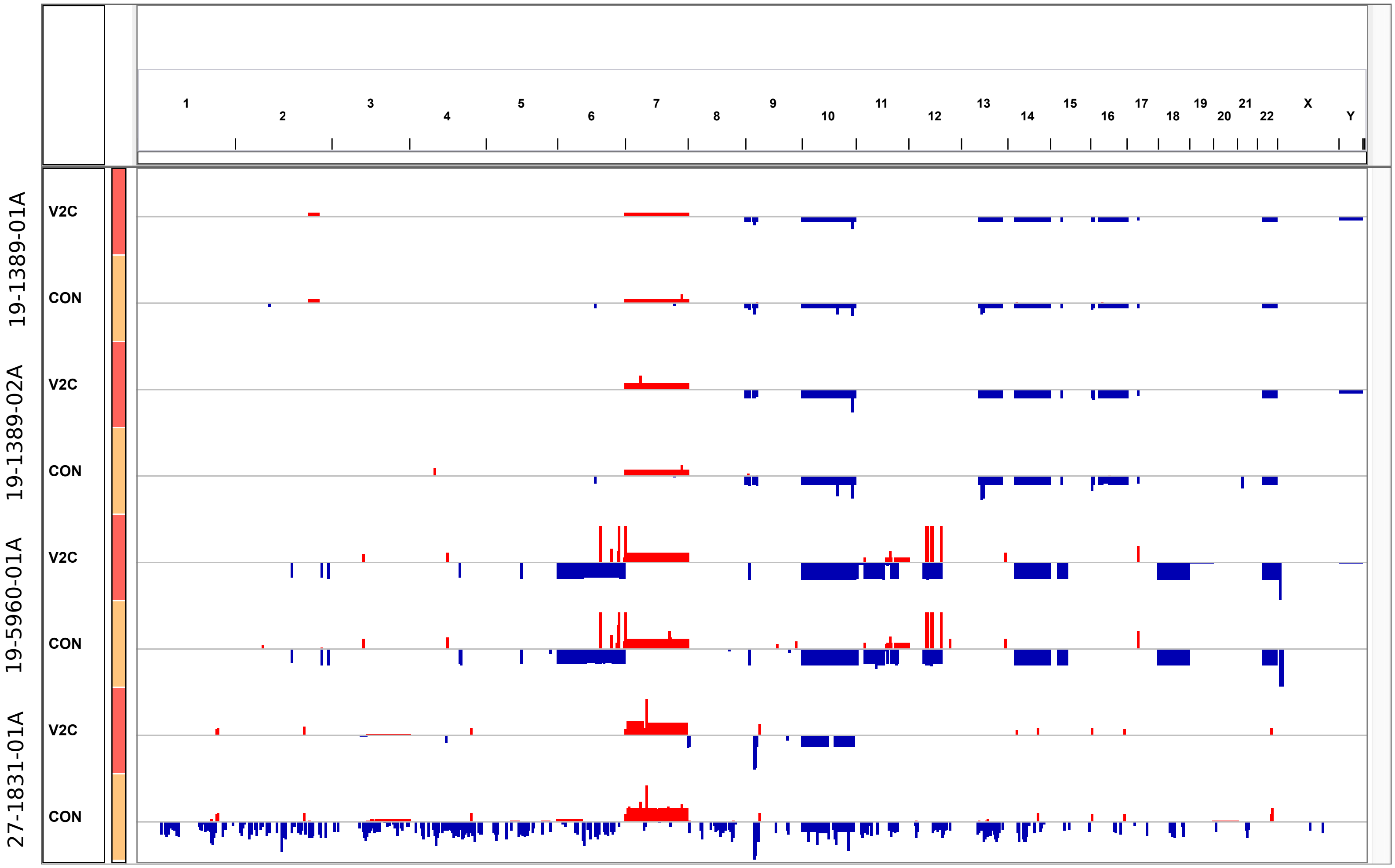

1
2
3
4
5
6
7
8
9
10
11
12
13
14
15
16
17
18
19
20
21
22
X
Y
19-1389-01A
V2C
CON
19-1389-02A
V2C
CON
19-5960-01A
V2C
CON
27-1831-01A
V2C
CON

S3:

A Circos plot of VCF2CNA (outer ring) and CONSERTING (inner ring), depicting high-amplitude focal CNA segments in 34 TCGA-GBM samples. A) 21 fracture genome TCGA-GBM samples. B) 13 previously reported samples. CNA range is specified for each sample

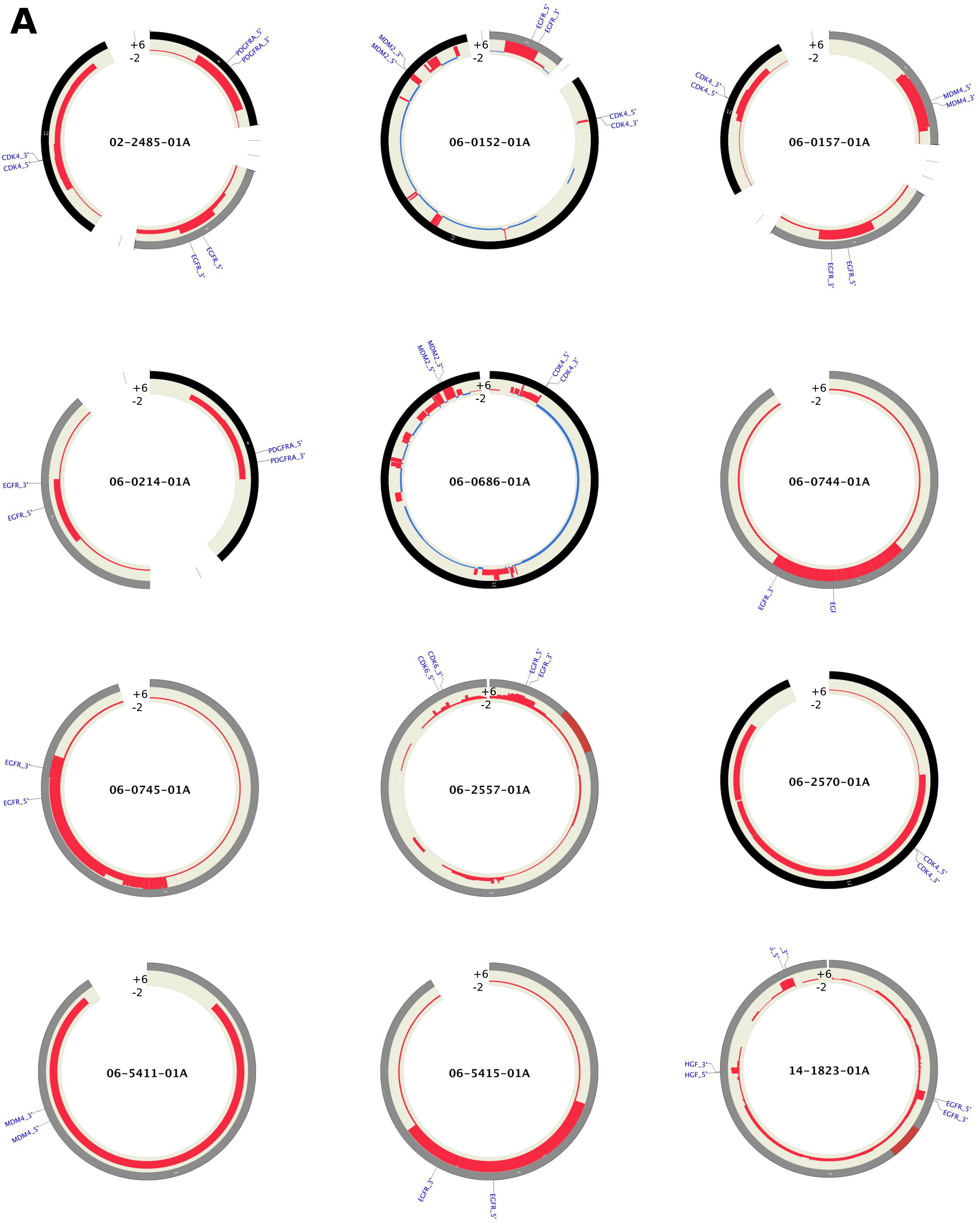

A Continued

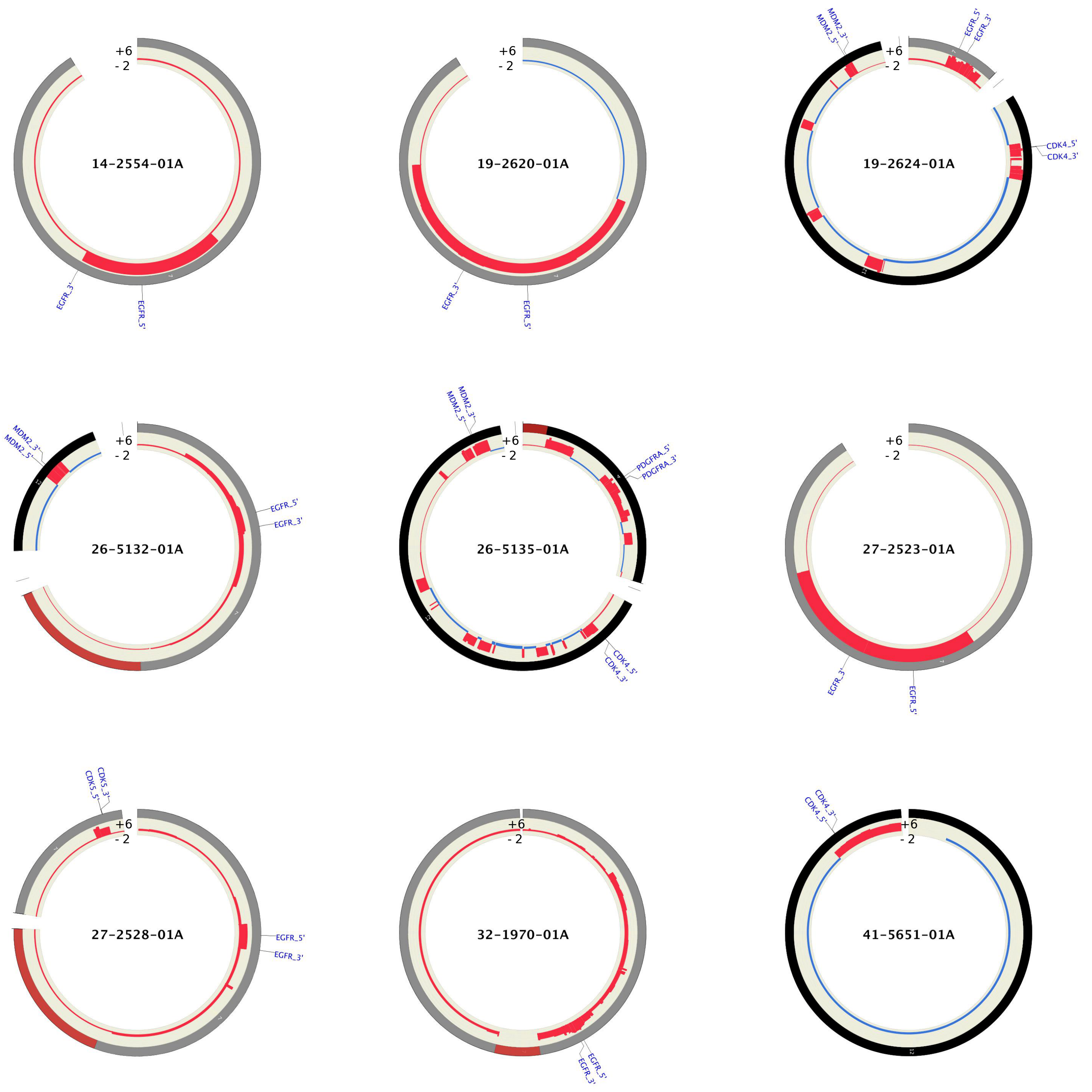
+6
- 2
14-2554-01A
EGFR_3'
EGFR_5'
19-2620-01A
19-2624-01A
MDM2_3'
MDM2_5'
EGFR_5'
EGFR_3'
CDK4_5'
CDK4_3'
26-5132-01A
26-5135-01A
PDGFRA_5'
PDGFRA_3'
CDK4_5'
CDK4_3'
27-2523-01A
27-2528-01A
CDK5_3'
CDK5_5'
32-1970-01A
41-5651-01A
CDK4_3'
CDK4_5'

B

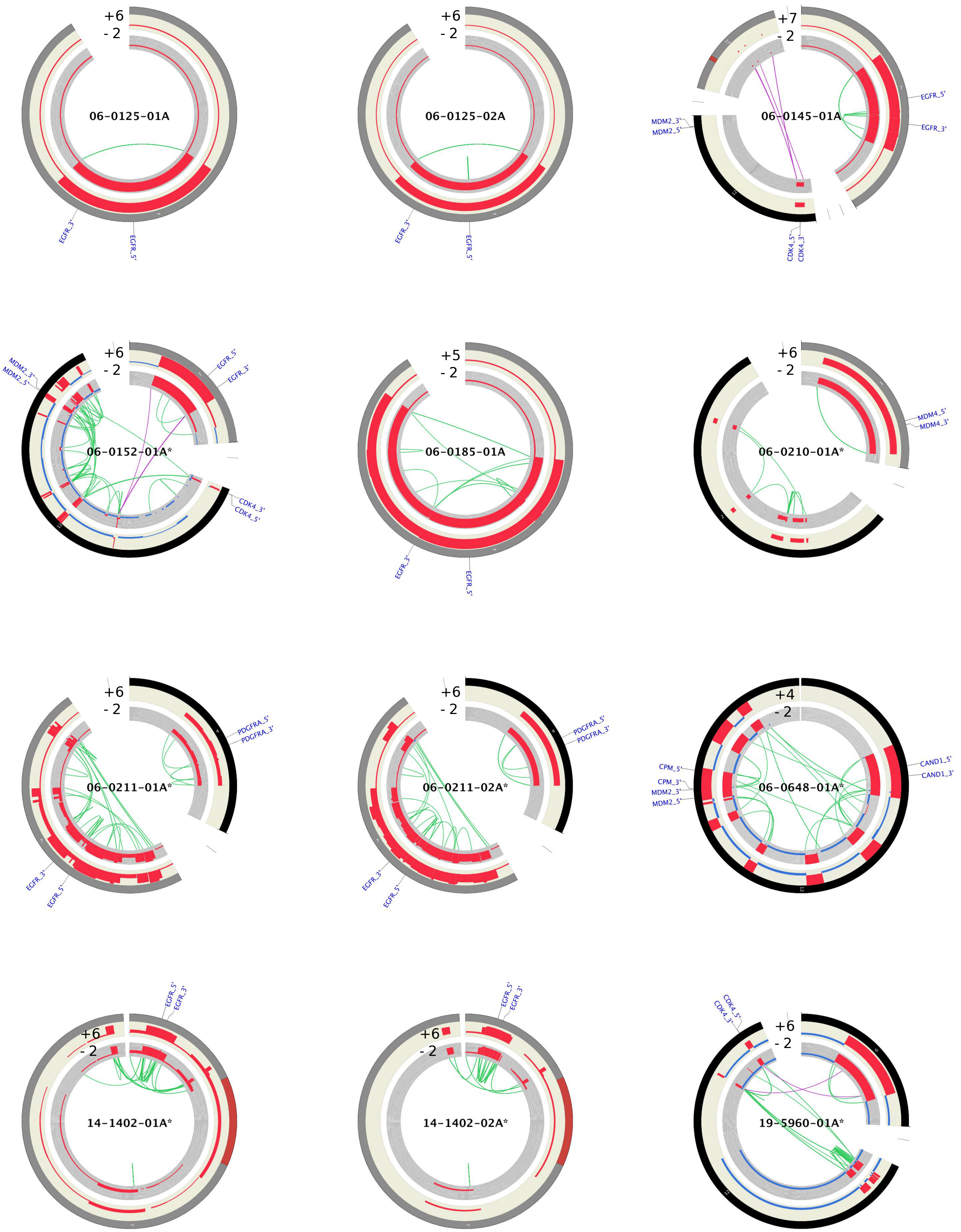
+6
- 2
06-0125-01A
EGFR_3'
EGFR_5'
+6
- 2
06-0125-02A
EGFR_3'
EGFR_5'
+7
- 2
06-0145-01A
EGFR_5'
EGFR_3'
MDM2_3'
MDM2_5'
CDK4_5'
CDK4_3'
+6
- 2
06-0152-01A*
MDM2_3'
MDM2_5'
EGFR_5'
EGFR_3'
CDK4_3'
CDK4_5'
+5
- 2
06-0185-01A
EGFR_3'
EGFR_5'
+6
- 2
06-0210-01A*
MDM4_5'
MDM4_3'
+6
- 2
06-0211-01A*
PDGFRA_5'
PDGFRA_3'
EGFR_3'
EGFR_5'
+6
- 2
06-0211-02A*
PDGFRA_5'
PDGFRA_3'
EGFR_3'
EGFR_5'
+4
- 2
06-0648-01A*
CPM_5'
CPM_3'
MDM2_3'
MDM2_5'
CAND1_5'
CAND1_3'
+6
- 2
14-1402-01A*
EGFR_5'
EGFR_3'
+6
- 2
14-1402-02A*
EGFR_5'
EGFR_3'
+6
- 2
19-5960-01A*
CDK4_5'
CDK4_3'

B Continued

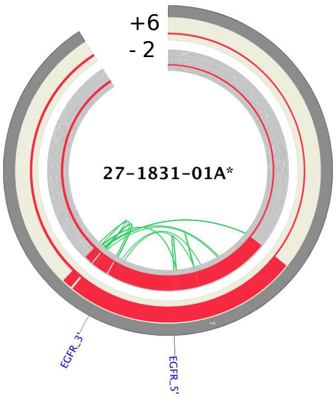

S4:

Segmental Overlap. A) A hypothetical large segment identified by CONSERTING (red) B) Subsequent focal segments identified by CONSERTING (blue). The original segment was split into five subsegments. None of the subsegments in b meet the reciprocal 50% segment overlap criteria with the original segment.

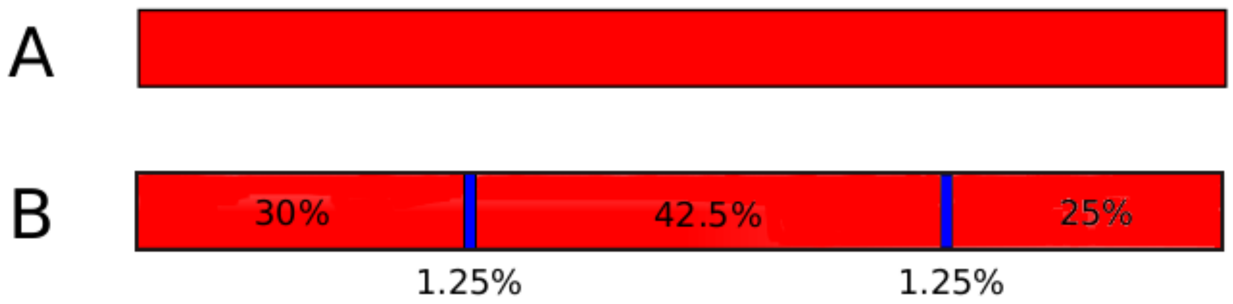

S5: Panel A

Circos plot of WXS (outer ring) and WGS (inner ring) for all 15 Rhabdomyosarcoma Xenograph samples. Legend depicts CNA range of data.

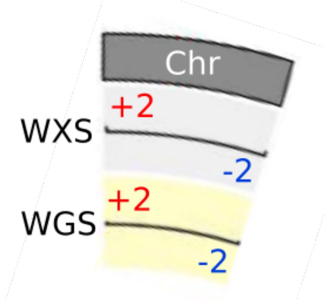

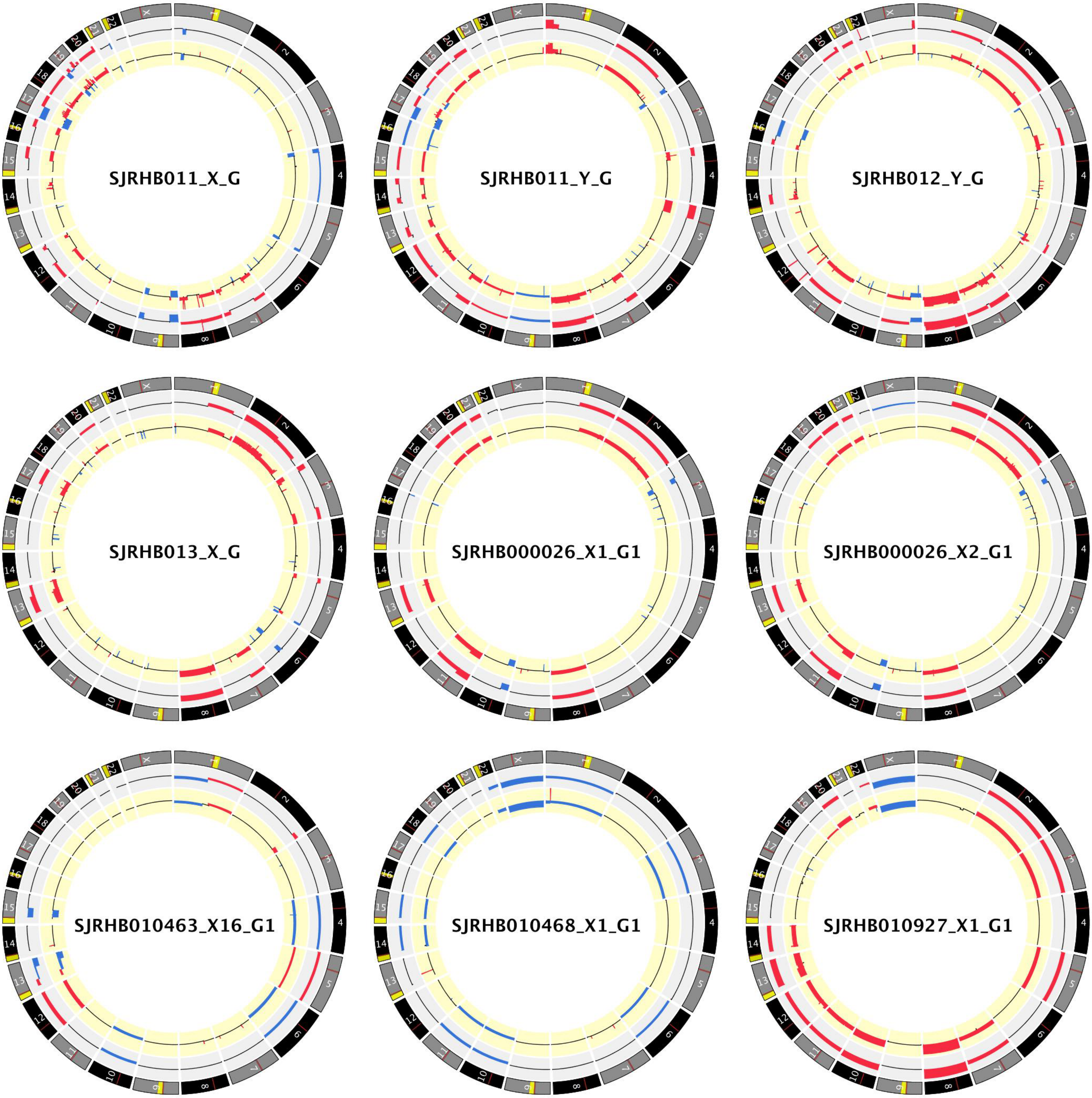

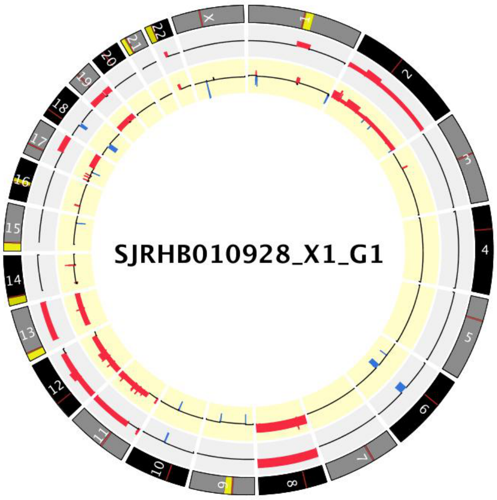
SJRHB010928_X1_G1

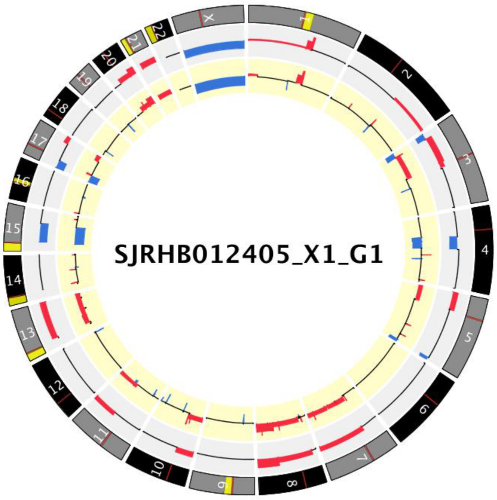
SJRHB012405_X1_G1

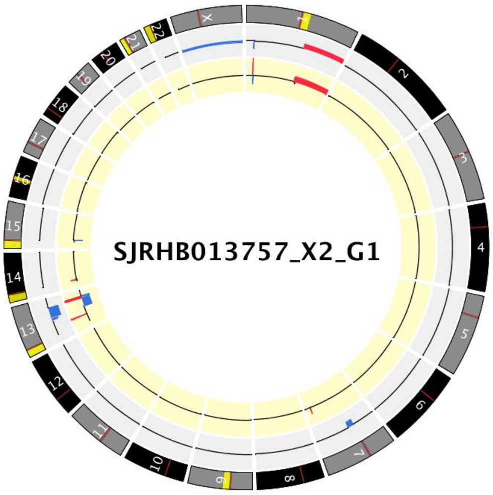
SJRHB013757_X2_G1

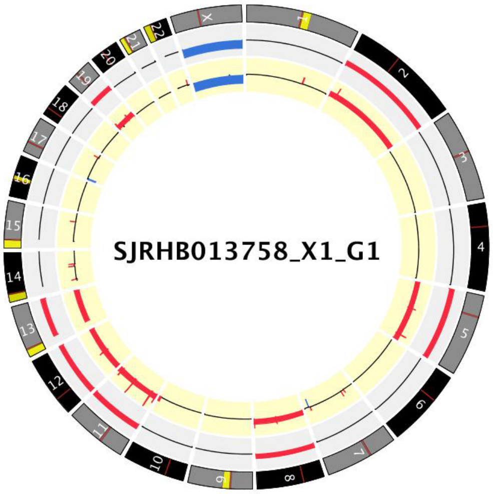
SJRHB013758_X1_G1

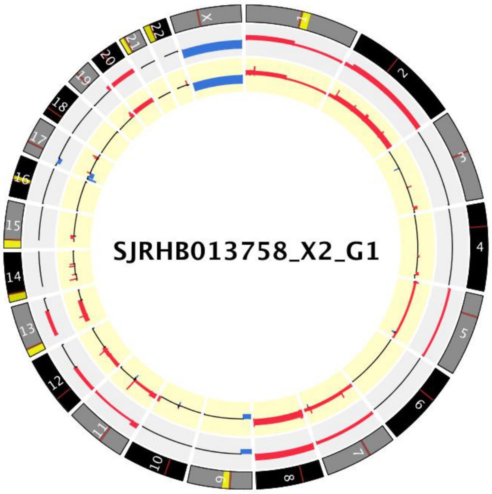
SJRHB013758_X2_G1

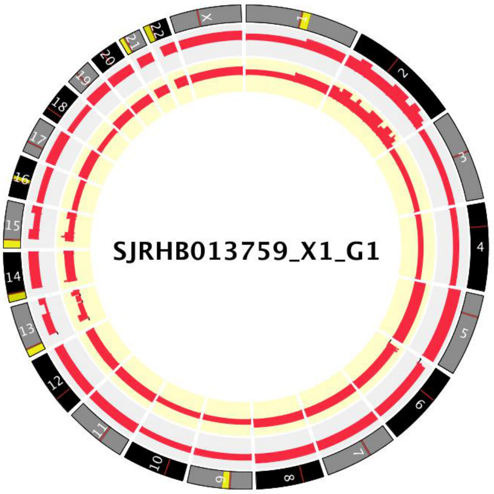
SJRHB013759_X1_G1

S5: Panel B

IGV plot of whole exome sequencing (WXS) and whole genome sequecing (WGS) for all 15 Rhabdomyosarcoma Xenograph samples.

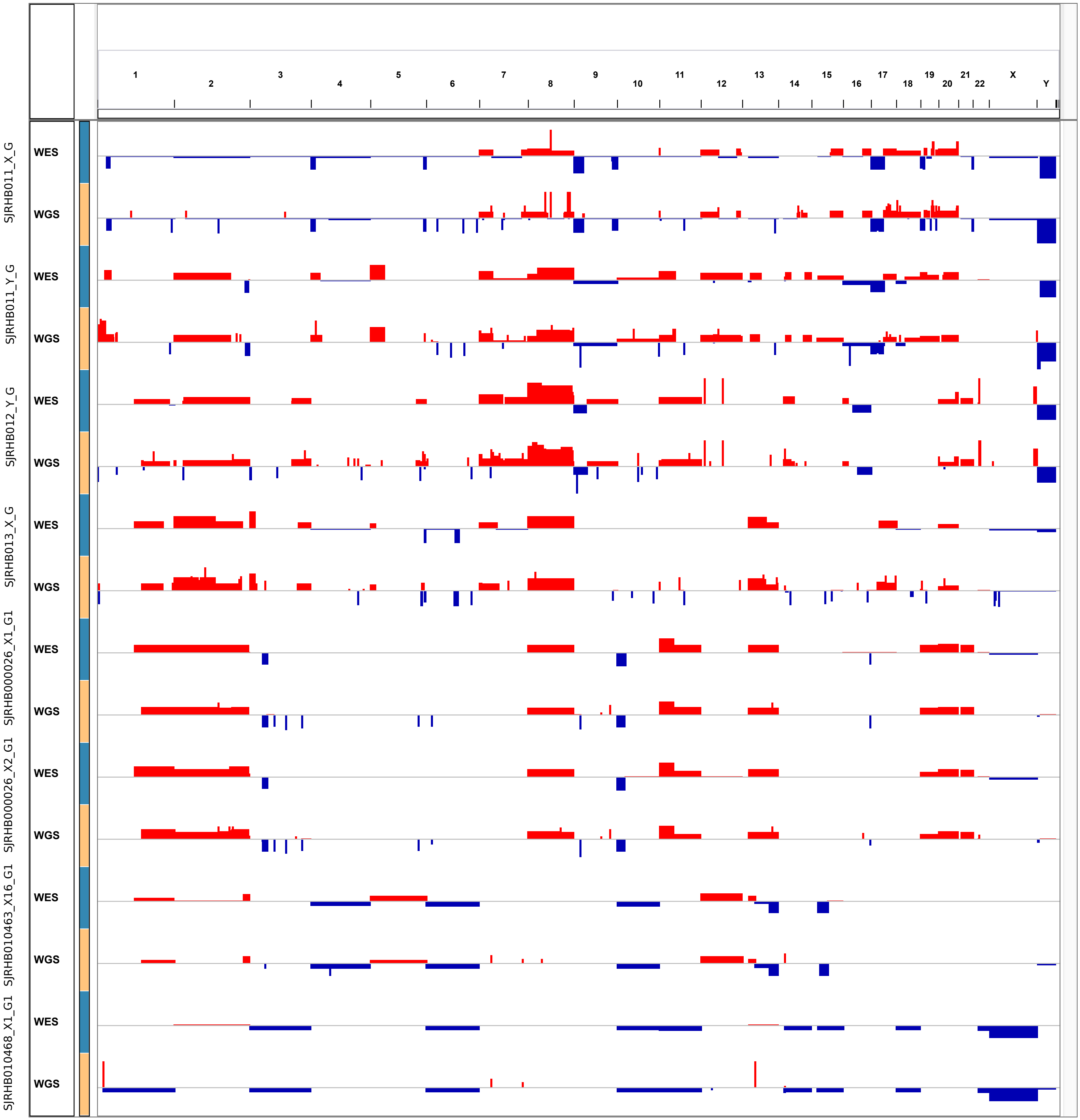

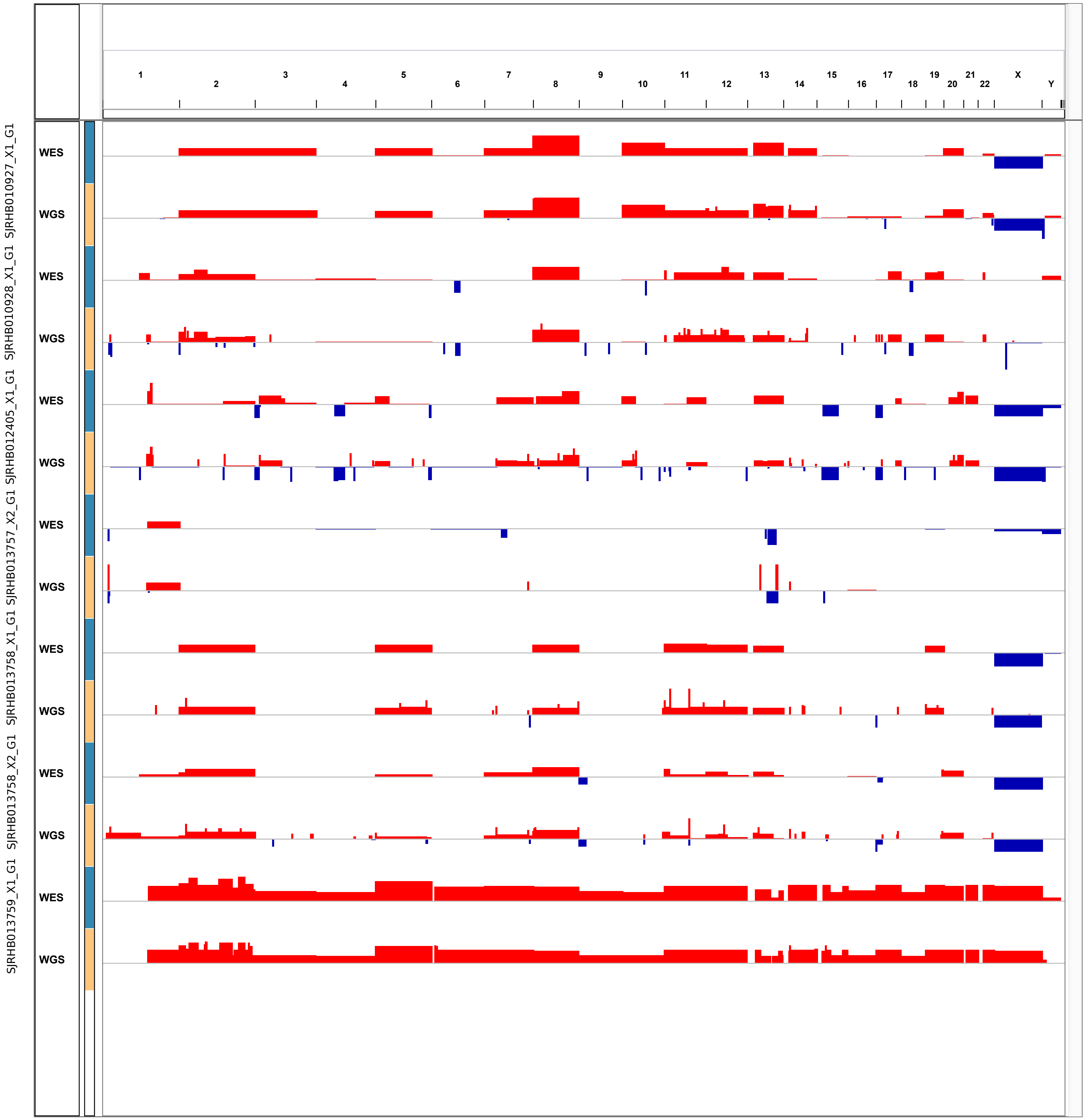

1
2
3
4
5
6
7
8
9
10
11
12
13
14
15
16
17
18
19
20
21
22
X
Y
SJRHB010927_X1_G1
SJRHB010928_X1_G1
SJRHB012405_X1_G1
SJRHB013757_X2_G1
SJRHB013758_X1_G1
SJRHB013758_X2_G1
SJRHB013759_X1_G1
WES
WGS
WES
WGS
WES
WGS
WES
WGS
WES
WGS
WES
WGS
WES
WGS

S6:
Density plot of SJRHB010468_X1_G1.

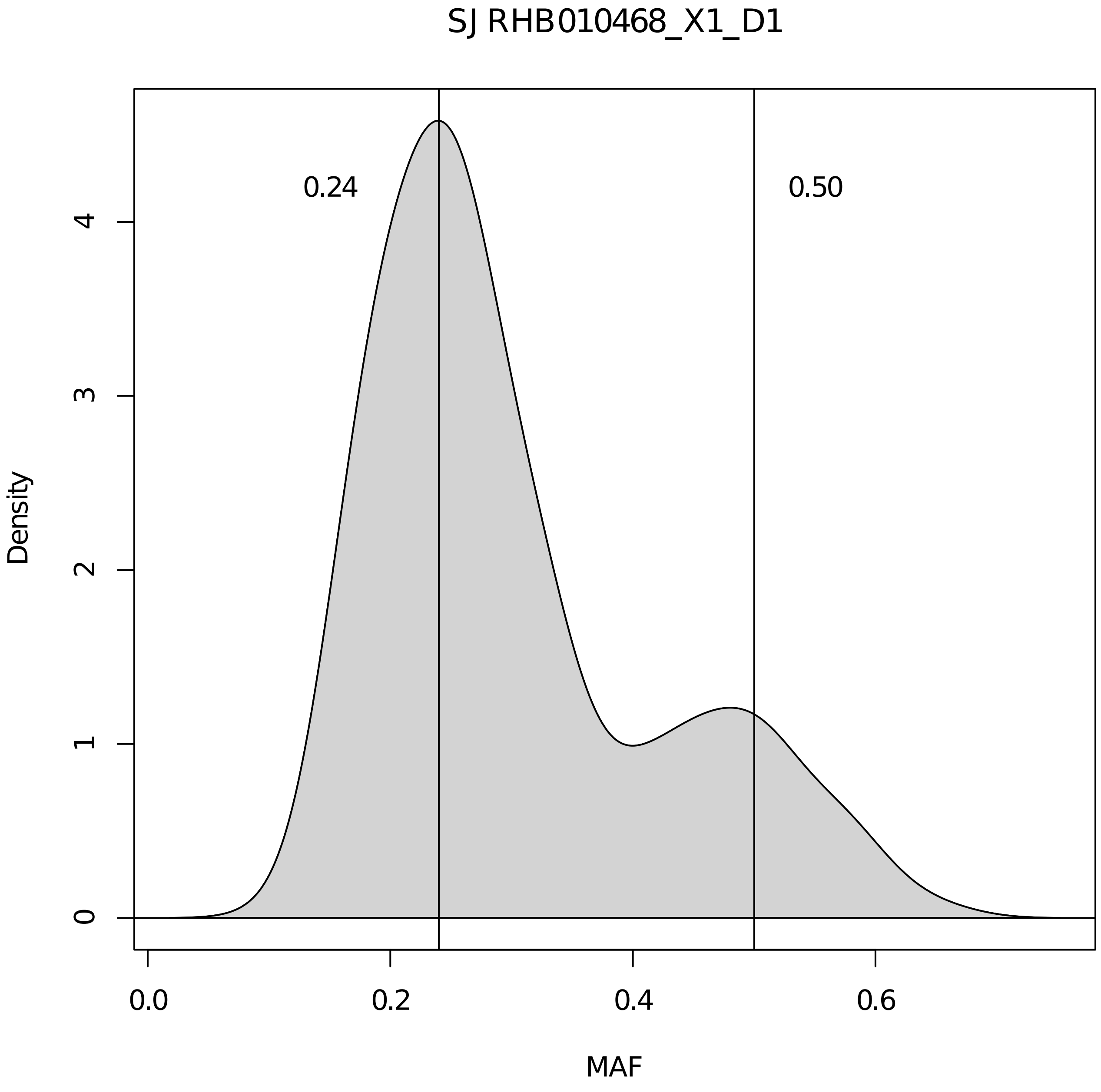

Supplement: Supplementary file 1 — Supplementary material [file 41598_2019_45938_MOESM1_ESM.pdf]
